# Narrative-based autobiographical memory interventions for PTSD: a meta-analysis of randomized controlled trials

**DOI:** 10.3389/fpsyg.2023.1215225

**Published:** 2023-09-27

**Authors:** Robert Raeder, Nicola S. Clayton, Markus Boeckle

**Affiliations:** ^1^Department of Psychology, University of Cambridge, Cambridge, United Kingdom; ^2^Scientific Working Group, Karl Landsteiner University of Health Sciences, Krems, Austria; ^3^Department of Transitory Psychiatry, University Hospital Tulln, Tulln, Austria

**Keywords:** narrative, PTSD, posttraumatic stress disorder, traumatic memories, autobiographical memory, memory

## Abstract

**Introduction:**

The aim of this systematic review and meta-analysis is to evaluate the efficacy of narrative-based interventions (NBIs) for individuals with post-traumatic stress disorder (PTSD). Investigating the efficacy of NBIs should yield insight on autobiographical memory (AM) phenomena implicated in PTSD onset and recovery, leading to improved intervention protocols. Furthermore, by analyzing how NBIs influence maladaptive AM distortions, we hope to shed light on the theorized narrative architecture of AM more generally.

**Methods:**

A systematic literature search was conducted according to PRISMA and Cochrane guidelines in MEDLINE, EMBASE, PsychINFO, and PubMed. Additional studies were then also identified from the reference lists of other relevant literature and considered for inclusion. Studies were then evaluated for adherence to the inclusion/exclusion criteria and assessed for risk of bias. Various meta-analyses were performed on included studies to understand how NBIs may or may not influence the overall effect size of treatment.

**Results:**

The results of the meta-analysis of 35 studies, involving 2,596 participants, suggest that NBIs are a viable and effective treatment option for PTSD, yielding a statistically significant within-group effect size and decrease in PTSD symptomatology at both post-treatment [*g* = 1.73, 95% CI (1.23–2.22)] and 3–9 month follow-up assessments [*g* = 2.33, 95% CI (1.41–3.26)]. Furthermore, the difference in effect sizes between NBIs compared to active and waitlist controls was statistically significant, suggesting that NBIs are superior. Sub-analyses showed that NET provided a stronger effect size than FORNET, which may be due to the nature of the traumatic event itself and not the treatment protocol. While evidence of small study and publication bias was present, a weight-function model and trim-and-fill method suggested it was not influencing the overall results.

**Discussion:**

This meta-analysis presents strong evidence supporting the use of NBIs in the treatment of PTSD. Clear similarities can be identified between NBIs included in this analysis that make them distinct from non-NBI interventions, which are reviewed in the discussion. Controlled comparisons between NBIs and non-NBIs would help to further understand AM mechanisms of action implicated in recovery and how various interventions facilitate them. Future research should also aim to elucidate the full range of AM impairment in individuals with PTSD to gain insight on how other memory capabilities, such as the ability to mentally simulate the future, are implicated in the pathogenesis of PTSD.

## Introduction

**Figure 1:**
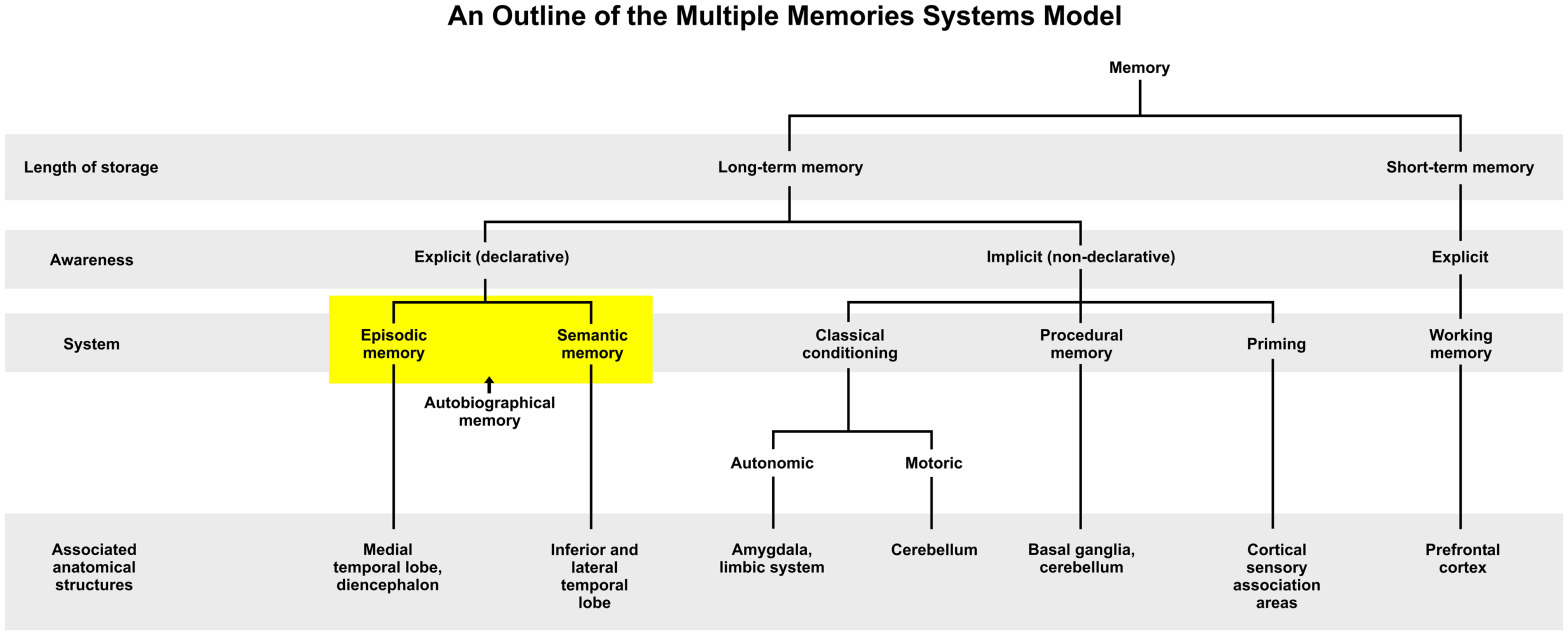
An outline of the Multiple Memory Systems Model (adapted by Raeder, 2022 from Schacter and Tulving, 1994; Willingham, 1997; Budson and Price, 2003; Ashby and O’Brien, 2005).

Autobiographical memory (AM) refers to an individual’s mental constructions of episodes or experiences that occurred in his or her own life, which is comprised of a blend of episodic memories (e.g., recalling a recent trip to Disney World) and semantic memories (e.g., remembering that Disney World is in Florida) (Tulving, 1972; Rubin, 1988; Conway, 1990, 1996; Conway and Rubin, 1993; Williams et al., 2008; see also “Mental Time Travel”; Suddendorf and Corballis, 1997). Episodic and semantic memories are both types of declarative memory identified in the multiple memory systems theory (MMS), which is a model of memory architecture that is largely supported by the neuroscientific literature (Figure 1; see Squire, 1992; Schacter and Tulving, 1994; Tulving and Craik, 2000; for autobiographical memory, Sheldon et al., 2019).

AM is dependent on the projection of self through various timescales, which Conway and Pleydell-Pearce’s (2000) theory described as a transitory “working self,” integrating an existing underlying knowledge base with new experiential information to link a personal past with a prospective future (also see Schacter and Addis, 2007). According to this theory, AM serves two main functions: adaptation to life events (e.g., an accurate record of experience to guide future-oriented behavior) and the assurance of a coherent sense of self (e.g., a conceptualization of one’s identity within his or her life story). In psychotherapy, the patient’s mentally narrated story is often the key focus of the healing process. By challenging the patient’s thoughts and beliefs about past memories, future projections, and self-identity, the psychotherapeutic conversation can help to restructure that inner monologue (Singer et al., 2013). Through this process, the patient is able to reauthor a different life story by finding a new perspective, thereby also restructuring incongruent autobiographical information (Singer and Blagov, 2004). In many ways, the clinician’s role in the psychotherapeutic process is to be both witness to the patient’s self-narrated story and “co-editor” of that unfolding narrative, offering helpful and evidence-based treatment when problems arise (Avdi and Georgaca, 2007).

Given the central role of AM in shaping an individual’s life narrative, it is not surprising that maladaptive AM distortions can lead to psychological distress, which is especially observable in post-traumatic stress disorder (PTSD). In cognitive models of PTSD, it is posited that onset is due to an inability to integrate new and traumatic experiential information with prior AM, thus resulting in a disruption to one’s overall life narrative or beliefs about oneself (Janoff-Bulman, 1992; Brewin et al., 1996; Ehlers and Clark, 2000). As such, it is well-established that AM distortions are a core symptom of PTSD and a critical component of the diagnostic criteria (The Diagnostic and Statistical Manual of Mental Disorders, 5th ed.; DSM-5; American Psychiatric Association, 2013). These distortions can include problems with coherence, content, intrusiveness, overgeneralization, specificity, veracity, vividness, voluntariness, as well as changes in self-perception and goal-pursuit (see Table 1 for a small sample of relevant literature).

**Table 1:** A brief review of relevant literature investigating maladaptive AM distortions in PTSD.

Type of AM distortion	Examples of relevant studies
Coherence	Follmer Greenhoot et al. (2013); Gray and Lombardo (2001); Halligan et al. (2003); Jaeger et al. (2014); Jelinek et al. (2009, 2010); Jones et al. (2007); Lindblom and Gray (2010); McNally (2003); Murray et al. (2002)
Consistency of content	Dekel and Bonanno (2013)
Event centrality	Brown et al. (2010)
Intrusiveness (Flashbacks)	Berntsen and Hall (2004)
Intrusive future simulations (Flashforwards)	Holmes et al. (2007); Loganovsky and Zdanevich (2013)
Goal pursuit	Jobson et al. (2014); Krans et al. (2017)
Overgeneralizations	Brown et al. (2013)
Self-perception	Sutherland and Bryant (2005); Kleim et al. (2007)
Specificity	Schönfeld and Ehlers (2017)
Veracity	Berntsen and Nielsen (2021)
Vividness	Berntsen et al. (2003)
Voluntariness	Catarino et al. (2015)

**Figure 2:**
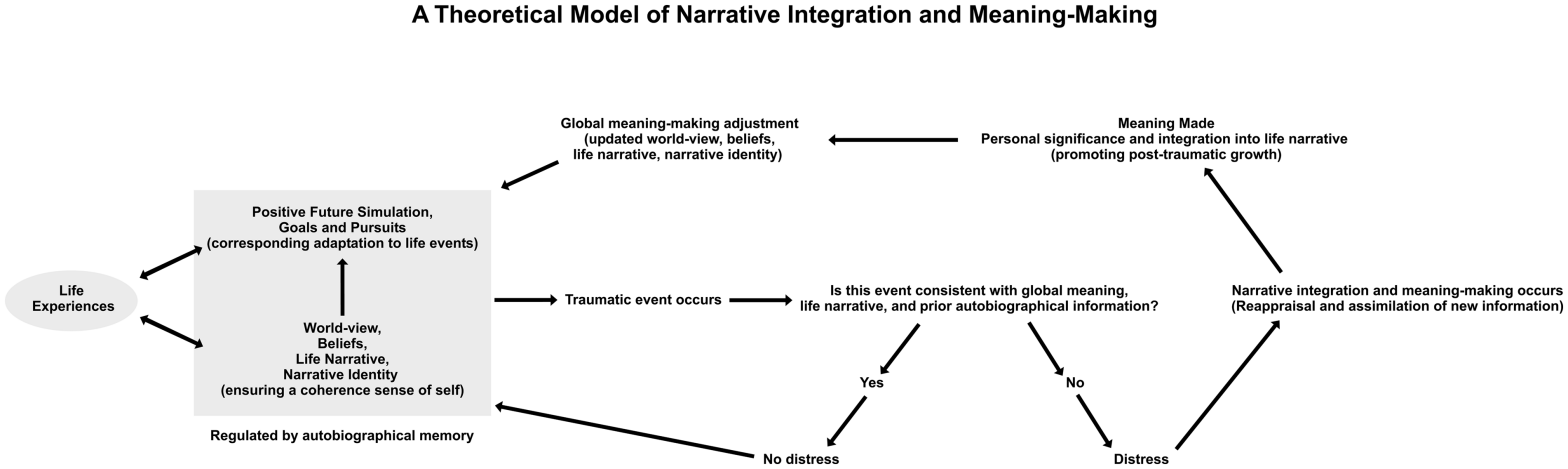
A theoretical model of narrative integration and meaning-making (adapted by Raeder, 2022 from Hartog et al., 2017; Park, 2022). In the figure, the grey box on the left represents the baseline or status quo for an individual regulating their sense of self and adaptation to life events through autobiographical memory. Upon experiencing a traumatic event, the individual will appraise whether or not novel information is congruent with prior autobiographical information. If congruent, then the individual is unlikely to experience distress and will thus return to baseline. However, if the traumatic experience is incongruent with prior autobiographical information, then the individual is likely to experience psychological distress and thus engage in a process of reappraisal and assimilation to make meaning from this new information in order to return to baseline.

The psychotherapeutic process of restructuring the patient’s mentally narrated story, outlined above, is therefore particularly important in the pathogenesis of PTSD, given its strong association with AM disturbances, but the mechanism which modulates this restructuring of maladaptive AM phenomena in the recovery process still remains unclear — most likely because the architecture of AM still requires further elucidation in a well-regulated model of mind. However, there is a wide body of literature indicating that “meaning-making” within the AM system is a key factor in the recovery process (e.g., Marin and Shkreli, 2019; Zakarian et al., 2019; Beierl et al., 2020; Huang et al., 2020; Fitzke et al., 2021; Park and Boals, 2021). According to Park (2010, 2022), meaning-making refers to an individual’s ability to orient oneself by making sense of experiential information, thus bringing potential global and situational discrepancies into congruence. Narrative integration and meaning-making models have also been used to accurately predict trajectories of distress and recovery following trauma; a process of reappraisal and assimilation in order to incorporate new and traumatic information into prior autobiographical information (Figure 2).

Outcomes of meaning-making from traumatic events can vary and range from individuals reporting a new understanding of why the event occurred (Beierl et al., 2020) to others feeling as though they have changed in a positive way because of their traumatic experience (Park and Boals, 2021). Some narrative-based interventions (NBIs) for PTSD, such as Narrative Exposure Therapy (NET) have also shown efficacy in the literature with appropriate effect size and statistical power, although the mechanisms modulating this effect are not fully understood (for reviews, see Gwozdziewycz and Mehl-Madrona, 2013; Lely et al., 2019b; Wei and Chen, 2021). As such, it remains unclear whether an observed improvement in narrative coherence within the patient’s life story through meaning-making is evidence of adjustment having occurred or if it is somehow implicated as a mechanism of therapeutic action that addresses maladaptive AM distortions. Importantly, notable parallels can be drawn between narrative meaning-making and other well-established psychological concepts, such as adaptation, acceptance, post-traumatic growth, and coping (Hartog et al., 2017). Additionally, there are many established and guideline-supported therapeutic interventions with strong evidence in the literature that may implicate the same recovery mechanisms of building meaningful coherence in the patient’s self-narrated life story, but a controlled comparison has not yet been carried out. These interventions include Cognitive Behavioral Therapy with a Trauma Focus (TF-CBT), Cognitive Processing Therapy (CPT), Cognitive Therapy (CT), Consolidation/Reconsolidation Therapy, Eye Movement Desensitization and Reprocessing (EMDR), Prolonged Exposure (PE), as well as adjunct pharmacological approaches, including MDMA- or psilocybin-assisted psychotherapy. This systematic review and meta-analysis therefore aims to move the field forward by critically examining the current body of research on PTSD interventions that specifically target maladaptive AM distortions through a narrative-based approach (NBIs) and to assess the efficacy of these interventions in reducing symptoms in individuals with a valid diagnosis of PTSD.

## Materials and methods

### Defining narrative-based interventions

For the purpose of this meta-analysis, a proposed definition of NBIs based on form and function is outlined in Table 2.

**Table 2:** A proposed rubric to define NBIs based on form and function.

Definition of narrative-based interventions	
Form	Narrative-based interventions can be creative/artistic [e.g., expressive writing or scrapbooking; see Garcia-Pelegrin et al. (2021) for theoretical reasoning] or a more specific and manualized protocol (e.g., NET or Narrative Reconstruction). Narrative-based writing protocols, such as expressive or creative writing about an experience or one’s life story more generally, are notably different from fact-focused or Socratic questioning writing protocols, such as the guidelines used in CPT for PTSD (Resick, 1992; Resick and Schnicke, 1992, 1993).
	Narrative-based interventions are often dynamic (e.g., there is not necessarily an “endpoint”), as integration and meaning-making from experiential information into a coherent life narrative is a constant and ever-changing process for each individual (Fivush et al., 2017). This dynamic quality is also in alignment with the reconstructive nature of AM, which is influenced by both an individual’s internalized story and how that story is nested within sociocultural stories writ large (Bartlett, 1932).
Function	Narrative-based interventions work to build coherence in autobiographical memories at both a local resolution (e.g., the most traumatic moment) and a global resolution (e.g., one’s life narrative).
	Narrative-based interventions aim to integrate traumatic memories into an individual’s life narrative in a meaningful way, a process of reappraisal and assimilation in order to incorporate new and traumatic information into prior autobiographical information. According to Hartog et al. (2017), this narrative integration process hinges on the ability to derive a narrative identity and meaning from the “randomness” of life events that may have conflicted with an individual’s previous worldview in order to form a coherent life narrative. Notably, the integration of meaning-making into one’s adjusted worldview is often achieved through narrative processing (e.g., the stories we tell ourselves and each other) and is aimed at reducing negative future contingencies while simultaneously embracing “new possibilities” due to the traumatic experience.

### Inclusion and exclusion criteria

Studies were accepted for consideration in this meta-analysis using the following inclusion criteria: (1) published in peer-reviewed journals or as part of a doctoral thesis; (2) recent and relevant (within the last 20 years); (3) randomized controlled trials (RCTs); (4) participants ages 15–85; (5) the majority of participants (at least 75%) met a valid diagnosis of PTSD at baseline according to the DSM-IV, DSM-5, as well as the ICD-9, ICD-10, and ICD-11; (6) use of valid clinician-administered or self-reported measurements of PTSD symptomatology; (7) the investigated intervention meets the aforementioned definition of narrative-based intervention (Table 2); (8) reported original data measuring PTSD symptoms pre- and post-treatment; (9) published in English or German. Studies were also included regardless of the context or “type” of trauma investigated.

Studies were excluded from consideration in this meta-analysis based on the following criteria: (1) participants under the age of 15 or over the age of 85; (2) participants with mental health comorbidities besides co-occurring anxiety and depression; (3) the usage of any other potentially confounding PTSD interventions; (4) lack of control or waitlist control in the study design.

### Search strategy

A search was conducted in MEDLINE, EMBASE, PsychINFO, and PubMed using medical subject headings (MeSH) and free text search term parameters (Table 3). Additional studies were also identified from the reference lists of relevant meta-analyses and other sources.

**Table 3:** Search parameters used in the initial literature search based on the Cochrane highly sensitive search strategies.

Database	Search parameters
Embase, MEDLINE, PsychInfo	(Exp narrative/ OR narration OR (narrative exposure therapy) OR (creative writing or written exposure therapy or expressive writing) OR (journaling OR journalling) OR (bibliotherapy) OR (scrapbooking) OR (imagery re-scripting) OR (narrative reconstruction) OR (story OR storytelling)) AND (exp Stress Disorders, Post-Traumatic/ OR (posttraumatic stress OR posttraumatic stress disorder* OR post-traumatic stress disorder* OR ptsd) OR (posttraumatic growth OR post-traumatic growth)) AND (Randomized controlled trial/ or Controlled clinical study/ or random$.ti,ab. or randomization/ or intermethod comparison/ or placebo.ti,ab. or (compare or compared or comparison).ti. or ((evaluated or evaluate or evaluating or assessed or assess) and (compare or compared or comparing or comparison)).ab. or (open adj label).ti,ab. or ((double or single or doubly or singly) adj (blind or blinded or blindly)).ti,ab. or double blind procedure/ or parallel group$1.ti,ab. or (crossover or cross over).ti,ab. or ((assign$ or match or matched or allocation) adj5 (alternate or group$1 or intervention$1 or patient$1 or subject$1 or participant$1)).ti,ab. or (assigned or allocated).ti,ab. or (controlled adj7 (study or design or trial)).ti,ab. or (volunteer or volunteers).ti,ab. or human experiment/ or trial.ti.) not (((random$ adj sampl$ adj7 (“cross section$” or questionnaire$1 or survey$ or database$1)).ti,ab. not (comparative study/ or controlled study/ or randomi?ed. controlled.ti,ab. or randomly assigned.ti,ab.)) or (Cross-sectional study/ not (randomized controlled trial/ or controlled clinical study/ or controlled study/ or randomi?ed. controlled.ti,ab. or control group$1.ti,ab.)) or (((case adj control$) and random$) not randomi?ed. controlled).ti,ab. or (Systematic review not (trial or study)).ti. or (nonrandom$ not random$).ti,ab. or “Random field$.”ti,ab. or (random cluster adj3 sampl$).ti,ab. or ((review.ab. and review.pt.) not trial.ti.) or (“we searched.”ab. and (review.ti. or review.pt.)) or “update review.”ab. or (databases adj4 searched).ab. or ((rat or rats or mouse or mice or swine or porcine or murine or sheep or lambs or pigs or piglets or rabbit or rabbits or cat or cats or dog or dogs or cattle or bovine or monkey or monkeys or trout or marmoset$1).ti. and animal experiment/) or (Animal experiment/ not (human experiment/ or human/)))
PubMed	(Narrative OR narration OR (“narrative exposure therapy”) OR (“creative writing” or “written exposure therapy” or expressive writing) OR (journaling OR journalling) OR (bibliotherapy) OR (scrapbooking) OR (“imagery re-scripting”) OR (“narrative reconstruction”) OR (story OR storytelling)) AND (exp Stress Disorders, Post-Traumatic/ OR (“posttraumatic stress” OR “posttraumatic stress disorder” OR “post-traumatic stress disorder” OR ptsd) OR (“posttraumatic growth” OR “post-traumatic growth”)) AND ((randomized controlled trial[pt]) OR (controlled clinical trial[pt]) OR (randomized[tiab] OR randomised[tiab]) OR (placebo[tiab]) OR (drug therapy[sh]) OR (randomly[tiab]) OR (trial[tiab]) OR (groups[tiab])) NOT (animals[mh] NOT humans[mh])

### Study selection process

**Figure 3:**
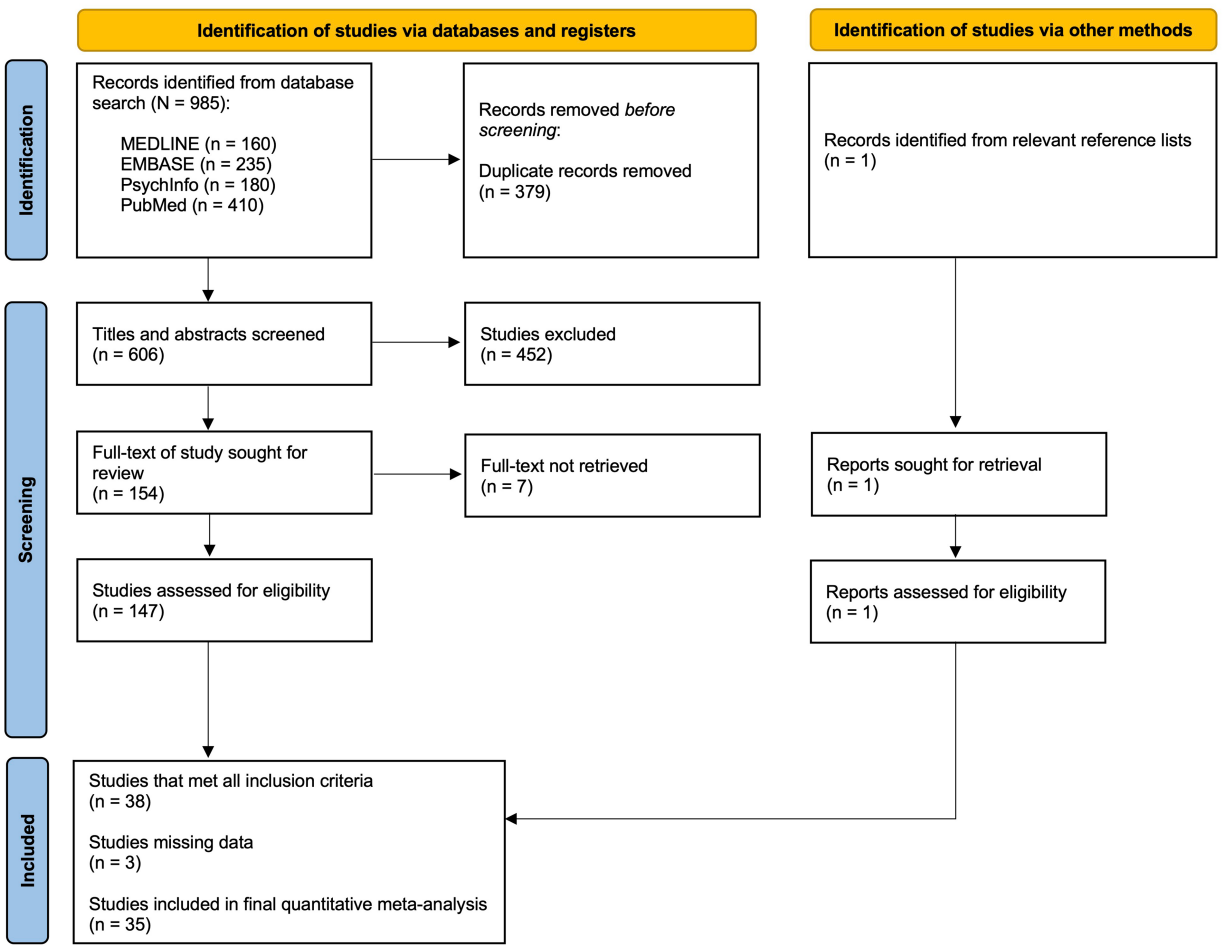
A PRISMA flow chart outlining the study selection process.

Figure 3 outlines a PRISMA flow chart detailing each stage of the exclusion process. The total database search results (*N* = 985) were first screened for duplicates. The remaining titles and abstracts (*n* = 606) were then screened for inclusion and exclusion criteria. Full-text articles (*n* = 154) were then sought to assess for eligibility; however (*n* = 7) studies were not accessible, even after making sufficient effort to contact the authors. One study (*n* = 1) was identified from the reference lists of relevant systematic reviews and meta-analyses that met the inclusion criteria. Ultimately, (*n* = 38) met full criteria to be included in this meta-analysis. However, there were 3 studies that met all inclusion criteria, but the full datasets from these studies were not accessible, even after adequate effort was made to reach out directly to all of the authors cited via email, ultimately resulting in their exclusion from our quantitative analysis. Finally, (*n* = 35) studies were included in the quantitative meta-analysis.

### Data extraction

Included studies were coded according to the following criteria: (1) active controls; (2) waitlist controls; (3) talk-therapy only interventions; (4) written-only interventions; (5) combination of talk-therapy and written interventions. We included data from baseline measurements, post-treatment assessments, as well as a follow-up assessment between 3 and 9 months if such data were measured and reported in the original studies via the same methodology as the post-treatment assessment. If more than one time point was assessed within the 3–9 month follow-up range, we included the latest reported data and excluded any in-between time points. For studies that reported follow-up assessments at 1 year or later, we recorded the data but did not include it in our analysis due to an overall lack of sufficient data from this longitudinal time interaction.

Assessments for PTSD diagnoses and symptomatology throughout the literature cited in this analysis follow the guidelines set forth in the DSM-IV, DSM-5, as well as the ICD-9, ICD-10, and ICD-11, and have shown excellent validity and reliability in the literature, including the PTSD Symptom Scale-Interview (PSS-I; Foa et al., 2016b), the Clinician-Administered PTSD Scale for DSM-5 (CAPS-5; Weathers et al., 2018), the PTSD Checklist (PCL; Blevins et al., 2015), the Posttraumatic Diagnostic Scale for DSM-5 (PDS-5; Foa et al., 2016a), the Structured Psychodiagnostic Interview for PTSD (SPTSS; Carlson, 2001), the Composite International Diagnostic Interview (CIDI; Quintana et al., 2012), the Harvard Trauma Questionnaire (HTQ; Mollica et al., 1992), the Davidson Trauma Scale (Davidson et al., 1997), the International Trauma Questionnaire (ITQ; Cloitre et al., 2018), the UCLA PTSD Index for DSM-5 (UPID; Kaplow et al., 2020), and the Impact of Event Scale-Revised (IES-R; Briere and Elliott, 1998; for Chinese version: Huang et al., 1992).

To be as conservative as possible and to improve the reliability of our results, we made the following adjustments: (1) for Al-Hadethe et al. (2015), we used the Emotional Freedom Therapy (EFT) intervention as the control group instead of the no treatment group; (2) for Neuner et al. (2004), we used the Supportive Counseling (SC) intervention as the control group instead of Psychoeducation (PE); (3) for Neuner et al. (2008), we used the Trauma Counseling (TC) intervention as the control group instead of the monitoring group; (4) for Sloan et al. (2022), we used the intent-to-treat data instead of the per-protocol data; (5) for McIntire (2014), we pooled the results from the trauma-assigned and the trauma-spontaneous writing groups; (6) for Zang et al. (2014), we pooled the results from the NET and the NET-Revised groups; (7) for Gray et al. (2017), we only used the results reported from the PSS-I assessment and excluded the results from the PCL-M assessment; (8) for Brady et al. (2021), we used the CAPS-5 measurements instead of the PCL-5; (9) for Womersley et al. (2020), we used the Cognitive Behavioral Therapy (CBT) group as the control instead of the waitlist group; (10) for Zang et al. (2013), the symptom scores from each symptom cluster were added within each group in order to derive the total IES-r mean score per the IES-r protocol (Horowitz et al., 1979) and then a corrected standard deviation was calculated by taking the square root of the sum of squared variances in each symptom cluster for each group using the following formula:


$$s=\sqrt{\frac{1}{n}}\overset{n}{\underset{i=1}{\sum }}{\left(x_{i}-\bar{x}\right)}^{2}$$

### Statistical analysis

In order to evaluate the efficacy of NBIs, a meta-analysis was performed using the “metafor” package, which was created for the R environment using standard methods (Viechtbauer, 2010). To assess the within-group effect size of NBIs, standardized mean differences (SMD) were calculated using the escalc() function, with the pre- and post-treatment sample sizes, mean PTSD symptom scores, and corresponding standard deviations as input variables in order to derive Hedges’ *g* (Hedges, 1981). The random-effects model was then calculated on the resulting standardized mean differences using the rma() function, with the calculated effect sizes and variances as input variables. Then, sub-analyses to assess the within-group effect size were performed using the same methods by first isolating NET interventions, as well as NET/FORNET (Forensic offender rehabilitation narrative exposure therapy) interventions. The decision was made to isolate NET/FORNET versus non-(NET/FORNET) into subgroups in order to compare the difference between the manualized protocol used in NET/FORNET with other included studies in which the intervention protocol was less structured. Additionally, as NET/FORNET constituted a large portion of the included studies, it also provided an opportunity to conduct a separate meta-analysis for this intervention protocol independently. The decision to then separate NET and FORNET in additional sub-analyses was made to isolate potentially incongruent trauma narratives in criminal offenders (FORNET) versus non-criminal offenders (NET) and to assess any differences in outcomes. Lastly, the within-group effect size of all NBIs was analyzed using the data from the 3–9 month follow-up assessments, adhering to the aforementioned within-group statistical protocol (SMD), in order to assess whether the effect size was sustained.

Next, to assess the between-group effect size, standardized mean differences were calculated using the escalc() function, with the pre- and post-treatment sample sizes, mean PTSD symptom scores, and corresponding standard deviations from both the NBI group, as well as the control group. The outputs of the escalc() functions were then used to create a data frame, subtracting the difference between the overall NBI effect size and the overall control effect size and adding the variances between the two groups. The variances were added because the combined effect size was calculated by taking the weighted average of the effect sizes, where the weights are the inverse of the variances. The random-effects model was then calculated on the resulting data frame using the rma() function, with the calculated effect sizes and variances as input variables. This analysis was performed comparing NBIs to active controls in all included studies and then in sub-analyses comparing NBIs to waitlist controls only. We then used the previously isolated NET, NET/FORNET, non-(NET/FORNET) sub-groups to assess between-group effect sizes for these interventions using the same methods.

Heterogeneity was assessed in each of the aforementioned statistical analyses using a Q statistic test, which is a chi-squared test that is used to determine whether the variation in the effect size between different studies is greater than what would be expected by chance. Our model also calculated the amount of total heterogeneity (tau^2^), the square root of the estimated variance (tau), the percentage of total heterogeneity (*I*^2^), and the total variability relative to sampling variability (*H*^2^). The tau^2^ statistic is a measure of the amount of variation among the studies included in the analysis, in which a higher tau^2^ value indicates more variation among the studies. The tau statistic is a measure of the standard deviation of the effect sizes among the studies included in the analysis, in which a higher value also indicates more variation among studies. The *I*^2^ statistic is a measure of how much of the total variability in the data is due to the variation between the studies included in the analysis, rather than due to sampling variability. A widely accepted interpretation of the *I*^2^ statistic is: 25% = low heterogeneity, 50% = moderate heterogeneity, and 75% = high heterogeneity (Higgins et al., 2003). The *H*^2^ statistic is a measure of the total variability in the data relative to the sampling variability, in which a higher value indicates more variability in the data. A leave-one-out analysis was used to test whether any singular omitted study would alter the pooled effect size or affect the overall heterogeneity. Finally, a Graphical Display of Study Heterogeneity (GOSH) plot was used to visualize the results of a combinatorial meta-analysis by plotting the summary of effect size estimates against the model’s heterogeneity utilizing the “ggplot2” package, which was also created for the R environment (Olkin et al., 2012; Wickham, 2016). A GOSH plot can help to identify potential sources of heterogeneity in the data and assess the impact of outlier subgroups on the overall results.

Small study and publication bias were assessed by creating a funnel plot, running Egger’s regression test and a rank correlation test. The funnel plot was used to visually display the effect sizes of different studies and the degree to which they are symmetrical around a central point. The regression test and rank correlation test were used to detect any systematic bias in the studies. As small study and publication bias was detected in the data, the Vevea and Hedges (1995) weight-function model was used from the “weightr” package to detect and address the bias effectively. The weight-function model estimates unadjusted fixed-, random-, and mixed-effects models, in which the effect sizes are normally distributed in order to assess and adjust for the presence of publication bias. Likewise, a trim-and-fill statistical method was used to correct for possible publication bias in the data and confirm the results of the weight-function model (Duval and Tweedie, 2000). This technique trims the confidence intervals of the studies that are most likely to be influenced by bias, and then fills in the gaps with imputed studies. Finally, risk of bias for all included studies was assessed using the revised Joanna Briggs Institute (JBI) critical appraisal tool for the assessment of risk of bias for RCTs (see Supplementary material; Barker et al., 2023).

## Results

Thirty-five studies, involving 2,596 participants, were included in the quantitative meta-analysis. Table 4 outlines the population subgroups, types of traumas, and other notable characteristics from all of the included studies in the present meta-analysis. The types of traumas investigated include fleeing as refugee and asylum seekers, political imprisonment, human trafficking, serious illness, torture, war and combat, intimate partner violence, loss of pregnancy, motor vehicle accidents, and surviving a natural disaster, among others. The types of NBIs assessed include NET, FORNET, Expressive Writing (EW), Narrative Reconstruction (NR), Reconsolidation of Traumatic Memories (RTM), Written Emotional Disclosure (WED), Narrative Writing (NW), Cognitive Narrative Therapy (CNT), and Written Exposure Therapy (WET). The identified populations among included studies indicate a wide geographic and cultural range, with patients from Saudi Arabia, Iraq, Germany, Portugal, Rwanda, Norway, Israel, China, North Korea, the United Kingdom, and the United States, among others, including various refugee and asylum seekers. Despite some included studies lacking a full range of demographic information, the overall mean age from the available data could be assessed as 35.45, with 48.67% of participants being male and 51.33% being female.

**Table 4:** Characteristics of included RCTs, treatment conditions, and primary measurements for PTSD symptoms.

Authors	Year	Population subtype	Trauma type	Age range	Mean age	Percentage male	Percentage female	Type of NBI	Talk or written therapy	Control	Study design	PTSD symptom measurement
Adenauer et al.	2011	Refugee and asylum seekers	Fleeing as refugee and asylum seekers	16–46	28.6	27.3	72.7	NET	Both	Waitlist	RCT	CAPS
Alessandri	2017	Undergraduate students	Various	NA	20.48	22.72	77.28	EW	Written	Directive protocol	RCT	PCL-S
Al-Hadethe et al.	2015	Iraqi students	Various	16–19	NA	100	0	NET	Both	EFT	RCT	SPTSS
Bichescu et al.	2007	Romanian political prisoners	Political imprisonment	NA	68.9	100	0	NET	Both	PED	RCT	CIDI
Brady et al.	2021	UK-residing survivors of trafficking	Human trafficking	NA	26.73	26.66	73.33	NET	Both	Waitlist	RCT	CAPS
Fan et al.	2021	Covid-19 patients in Xiangyang City	Hospitalized with COVID-19	NA	46.16	39.29	60.71	NET	Both	TAU	RCT	PCL-C
Gensichen et al.	2022	German adults	Critical illness in ICU	18–85	NA	NA	NA	NET	Both	TAU	RCT	PDS-5
Gofman et al.	2021	Israeli adults	Various	18–70	38.6	43.33	56.66	NR	Both	Waitlist	RCT	CAPS
Gray et al.	2017	US veterans	War and combat	NA	48.6	100	0	RTM	Talk	Waitlist	RCT	PCL-M; PSS-I
Hensel-Dittmann et al.	2011	Asylum seekers in Germany	War, combat, and torture	NA	NA	NA	NA	NET	Both	SIT	RCT	CAPS
Hermenau et al.	2013	Former child soldiers in Eastern DRC	War and combat (child soldier)	16–25	19	100	0	FORNET	Both	TAU	RCT	PSS-I
Hijazi et al.	2014	Iraqi refugees in Michigan	Fleeing as refugee and asylum seekers	NA	48.2	44.4	55.6	NET	Both	Waitlist	RCT	HTQ
Ironson et al.	2013	People with HIV in Florida	HIV diagnosis	18–70	42.8	60	40	WED	Written	FFW	RCT	Davidson
Jacob et al.	2014	Rwandan citizens	Rwandan genocide	NA	48.29 / 25.06	NA	NA	NET	Both	Waitlist	RCT	PSS-I; CAPS
Koebach et al.	2021	Former child soldiers in Eastern DRC	War and combat (child soldier)	16–75	33	NA	NA	FORNET	Both	TAU	RCT	PSS-I
Lely et al.	2019a	Older Dutch patients with PTSD	Various	55–81	62.65	72.2	27.8	NET	Both	PCT	RCT	CAPS
McIntire	2014	University students	Various	NA	19.67	76.66	23.33	NW	Written	Innocuous writing	RCT	CAPS; PCL
Morath et al.	2014a	Refugees with PTSD	War and torture	15–46	30	41.17	58.83	NET	Both	Waitlist	RCT	CAPS
Morath et al.	2014b	Refugees with PTSD	War and torture	15+	28.7	68.43	31.57	NET	Both	Waitlist	RCT	CAPS
Moreira et al.	2020	Females in Portugal	Intimate partner violence	18+	37	0	100	CNT	Talk	TAU	RCT	ITQ
Neuner et al.	2004	African refugees	Fleeing as refugee and asylum seekers	NA	31.9	46.7	53.3	NET	Both	SC	RCT	CIDI
Neuner et al.	2008	African refugees	Fleeing as refugee and asylum seekers	NA	34.4	49.5	50.5	NET	Both	TC	RCT	PDS
Neuner et al.	2009	Asylum seekers in Germany	Fleeing as refugee and asylum seekers	NA	31.1	68.8	31.2	NET	Both	TAU	RCT	PDS
Orang et al.	2018	Iranian women	Intimate partner violence	16–60	NA	0	100	NET	Both	TAU	RCT	PSS-I
Park et al.	2020	Refugees from North Korea	Fleeing as refugee and asylum seekers	16–24	18.89	33.33	66.66	NET	Both	TAU	RCT	UPID
Qian et al.	2021	Pregnant women	Termination of pregnancy	NA	30.06	0	100	EW	Written	TAU	RCT	IES-R
Robjant et al.	2019	Former child soldiers in Eastern DRC	War and combat (child soldier)	16–25	18	0	100	FORNET	Both	TAU	RCT	PSS-I
Sloan et al.	2011	University students	Various	NA	18.9	NA	NA	WED	Written	FFW	RCT	PSS-I
Sloan et al.	2012	Adults in Boston	Surviving a motor vehicle accident	18–65	40.65	35	65	WET	Written	Waitlist	RCT	CAPS
Sloan et al.	2018	Treatment seeking adults in Boston	Various	18+	43.86	52.38	47.62	WET	Written	CPT	RCT	PCL-5; CAPS-5
Sloan et al.	2022	Military service members in Texas	Various	18+	35	81.2	18.8	WET	Written	CPT	RCT	CAPS
Womersley et al.	2020	Men in South Africa	Chronic gang violence	16–40	23.4	100	0	FORNET	Both	CBT	RCT	PSS-I
Zang et al.	2013	Adult survivors of the Sichuan earthquake	Natural disaster	37–75	55.7	22.72	77.28	NET	Both	Waitlist	RCT	IES-R; PDS
Zang et al.	2014	Adult survivors of the Sichuan earthquake	Natural disaster	28–80	53.63	10	90	NET	Both	Waitlist	RCT	IES-R; PDS
Zolfa et al.	2022	Iranian women	Breast cancer	NA	41.09	0	100	WET	Written	TAU	RCT	PCL-5

NET, Narrative Exposure Therapy; EW, Expressive writing; NR, Narrative reconstruction; RTM, Reconsolidation of traumatic memories; FORNET, Forensic offender rehabilitation narrative exposure therapy; WED, Written emotional disclosure; NW, Narrative writing; CNT, Cognitive narrative therapy; WET, Written exposure therapy; EFT, Emotional freedom technique; PED, Psychoeducation; TAU, Treatment as usual; SIT, Stress inoculation training; FFW, Fact-focused writing; PCT, Present centered therapy; SC, Supportive counselling; TC, Trauma counselling; CPT, Cognitive processing therapy; PSS-I, PTSD Symptom Scale-Interview; CAPS, the Clinician-Administered PTSD Scale (−5 for DSM-5); PCL, the PTSD Checklist (−5 for DSM-5; −M for military specific assessment protocol); PDS-5, Posttraumatic Diagnostic Scale for DSM-5; SPTSS, the Structured Psychodiagnostic Interview for PTSD; CIDI, the Composite International Diagnostic Interview; HTQ, the Harvard Trauma Questionnaire; Davidson, the Davidson Trauma Scale; ITQ, the International Trauma Questionnaire; UPID, the UCLA PTSD Index for DSM-5; IES-R, the Impact of Event Scale-Revised.

Various statistical analyses yielded the following results (for a summary, see Table 5):

**Table 5:** A summary of results.

Level of analysis	Effect size (Hedge’s *g*)
NBI within-group analysis	1.73
NET-only within-group analysis	1.68
NET/FORNET within-group analysis	1.58
Non-(NET/FORNET) within-group analysis	2.01
NBI within-group analysis (3–9 month follow-up)	2.33
NBI between-group analysis (active controls)	0.57
NBI between-group analysis (waitlist controls)	1.93
NET-only between-group analysis (active controls)	0.79
NET-only between-group analysis (waitlist controls)	1.55
NET/FORNET between-group analysis (active controls)	0.78

All within-group analyses report the effect size from the pre- to post-treatment assessment measurements, except for the 3–9 follow-up results as indicated in the table. For the between-group analyses, the effect size is reported as the mean difference in Hedge’s *g* between the NBI and control groups.

### NBI within-group analysis

**Figure 4:**
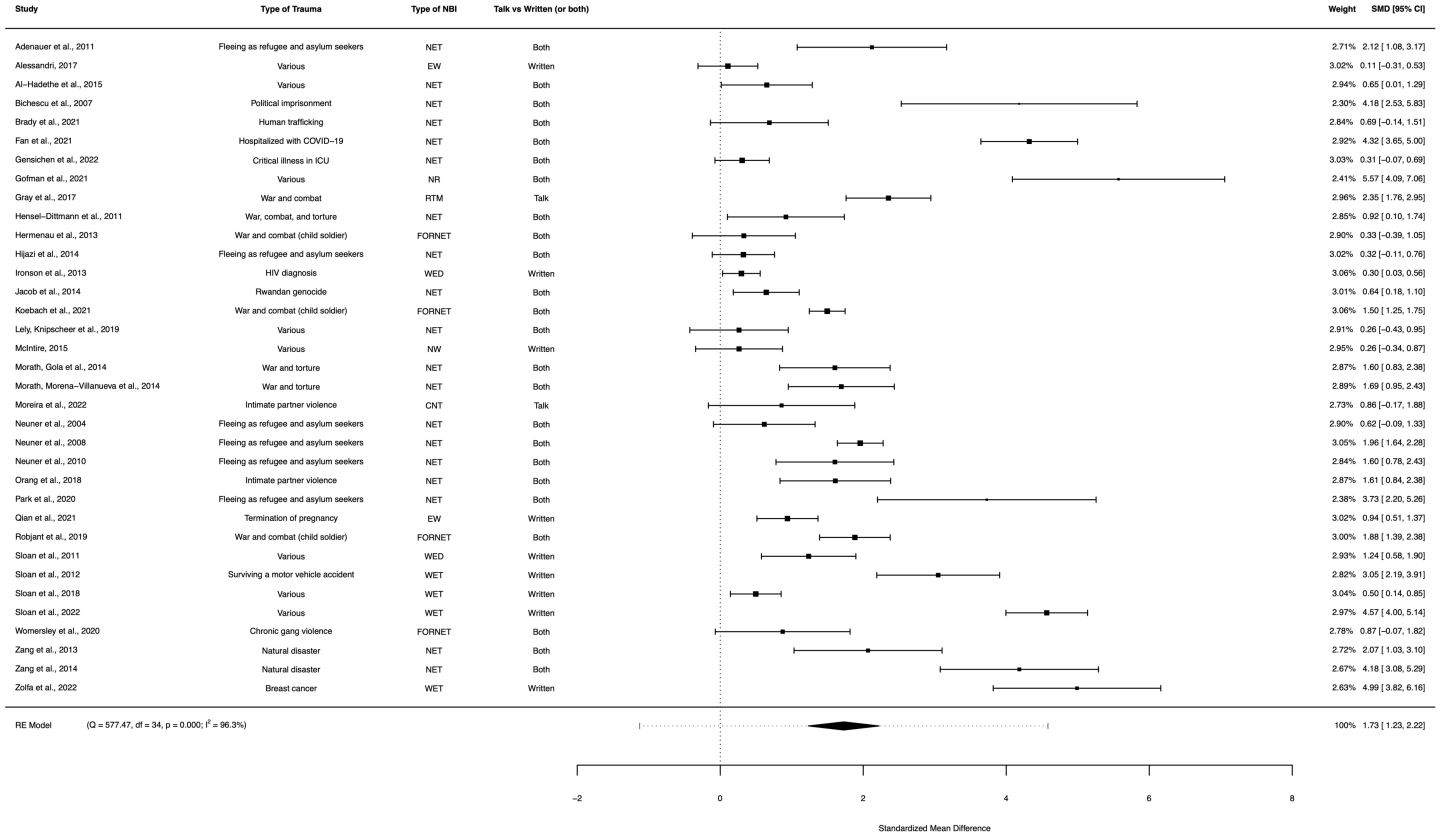
A forest plot indicating the within-group effect size of NBIs from pre- to post-treatment; NET, Narrative Exposure Therapy; EW, Expressive writing; NR, Narrative reconstruction; RTM, Reconsolidation of traumatic memories; FORNET, Forensic offender rehabilitation narrative exposure therapy; WED, Written emotional disclosure; NW, Narrative writing; CNT, Cognitive narrative therapy; WET, Written exposure therapy.

A within-group meta-analysis of NBIs (35 studies) from pre- to post-treatment indicated that NBIs were effective in the reduction of PTSD symptoms. The calculated standardized mean difference yielded a statistically significant pooled effect size of 1.73 (95% CI: 1.23–2.22, *p* < 0.0001; Figure 4). The total amount of heterogeneity in the studies, or tau^2^, was estimated to be 2.06 (*SE* = 0.54). The *I*^2^ statistic was calculated to be 96.31%, indicating a high degree of between study heterogeneity. The *H*^2^ statistic, which quantifies the ratio of total variability to sampling variability, was 27.13. The test for heterogeneity was highly significant [*Q*(*df* = 34) = 577.47, *p* < 0.0001].

### Net-only within-group analysis

**Figure 5:**
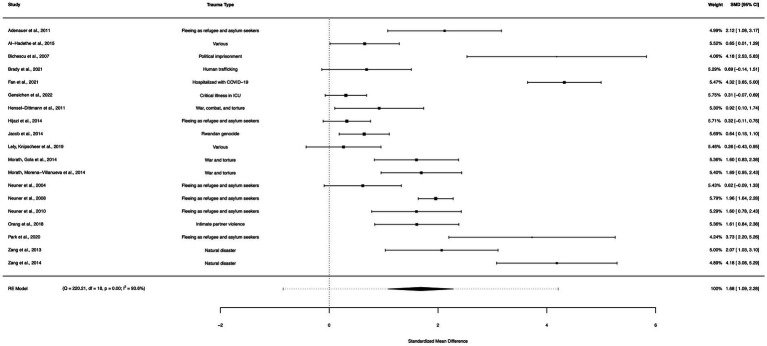
A forest plot indicating the within-group effect size of NET from pre- to post-treatment; NET, Narrative Exposure Therapy.

A within-group meta-analysis of NET-only interventions (19 studies) from pre- to post-treatment indicated that NET was effective in the reduction of PTSD symptoms. The calculated standardized mean difference yielded a statistically significant pooled effect size of 1.68 (95% CI: 1.09–2.28, *p* < 0.0001; Figure 5). The total amount of heterogeneity in the studies, or tau^2^, was estimated to be 1.57 (*SE* = 0.58). The *I*^2^ statistic was calculated to be 93.61%, indicating a high degree of between study heterogeneity. The *H*^2^ statistic, which quantifies the ratio of total variability to sampling variability, was 15.66. The test for heterogeneity was highly significant [*Q*(*df* = 18) = 220.21, *p* < 0.0001].

### Net/FORNET within-group analysis

**Figure 6:**
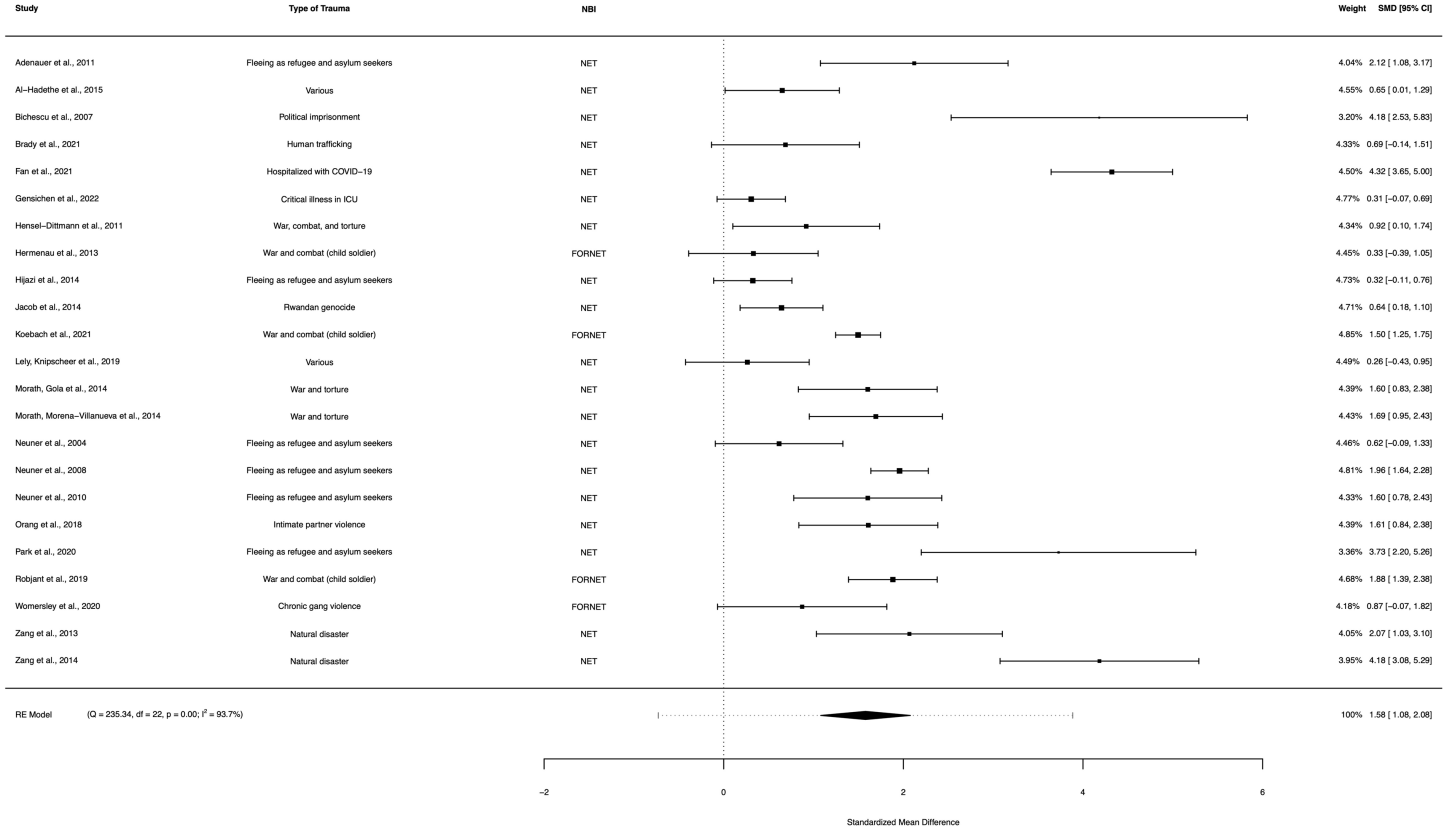
A forest plot indicating the within-group effect size of NET/FORNET from pre- to post-treatment; NET, Narrative Exposure Therapy; FORNET, Forensic offender rehabilitation narrative exposure therapy.

A within-group meta-analysis of NET/FORNET-only interventions (23 studies) from pre- to post-treatment indicated that NET/FORNET interventions were effective in the reduction of PTSD symptoms. The calculated standardized mean difference yielded a statistically significant pooled effect size of 1.58 (95% CI: 1.08–2.08, *p* < 0.0001; Figure 6). The total amount of heterogeneity in the studies, or tau^2^, was estimated to be 1.32 (*SE* = 0.45). The *I*^2^ statistic was calculated to be 93.70%, indicating a high degree of between study heterogeneity. The *H*^2^ statistic, which quantifies the ratio of total variability to sampling variability, was 15.87. The test for heterogeneity was highly significant [*Q*(*df* = 22) = 235.34, *p* < 0.0001].

### Non-(Net/FORNET) within-group analysis

**Figure 7:**
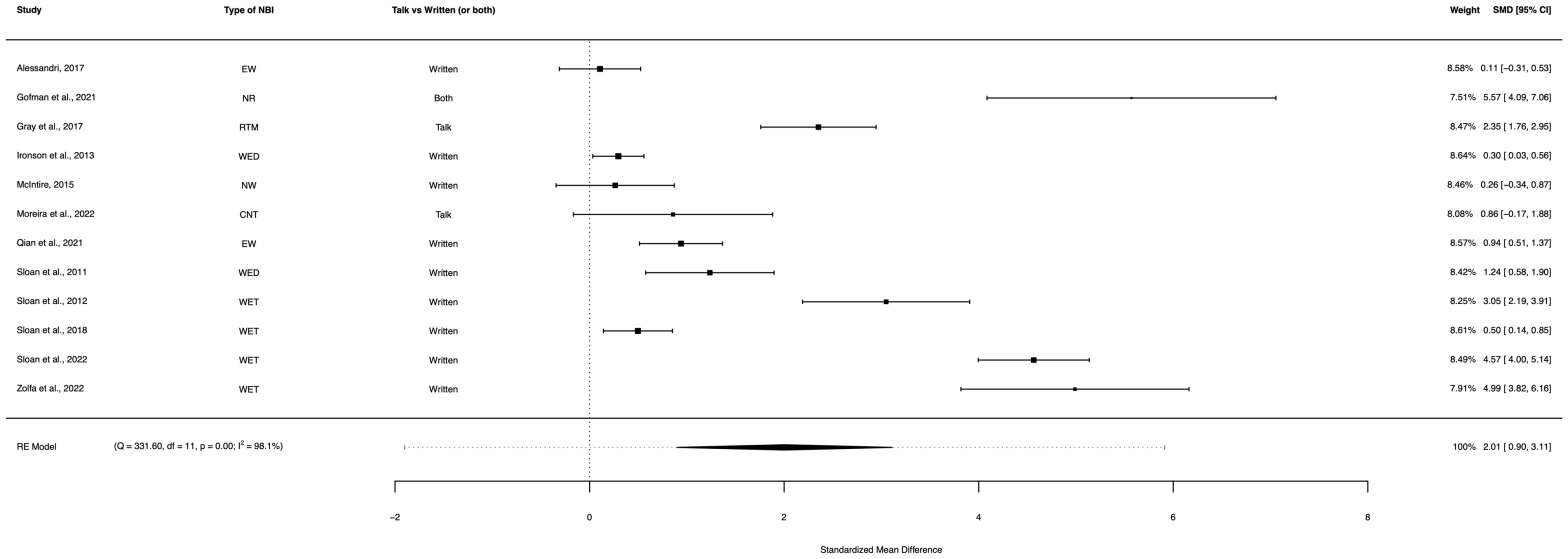
A forest plot indicating the within-group effect size of non-(NET/FORNET) from pre- to post-treatment; EW, Expressive writing; NR, Narrative reconstruction; RTM, Reconsolidation of traumatic memories; WED, Written emotional disclosure; NW, Narrative writing; CNT, Cognitive narrative therapy; WET, Written exposure therapy.

A within-group meta-analysis of non-(NET/FORNET)-only interventions (12 studies) from pre- to post-treatment indicated all remaining NBIs from this analysis (excluding NET/FORNET) were effective in the reduction of PTSD symptoms. The calculated standardized mean difference yielded a statistically significant pooled effect size of 2.01 (95% CI: 0.90–3.11, *p* < 0.0001; Figure 7). The total amount of heterogeneity in the studies, or tau^2^, was estimated to be 3.66 (*SE* = 1.62). The *I*^2^ statistic was calculated to be 98.10%, indicating a high degree of between study heterogeneity. The *H*^2^ statistic, which quantifies the ratio of total variability to sampling variability, was 52.52. The test for heterogeneity was highly significant [*Q*(*df* = 11) = 331.60, *p* < 0.0001].

### NBI within-group analysis (3–9 month follow-up)

**Figure 8:**
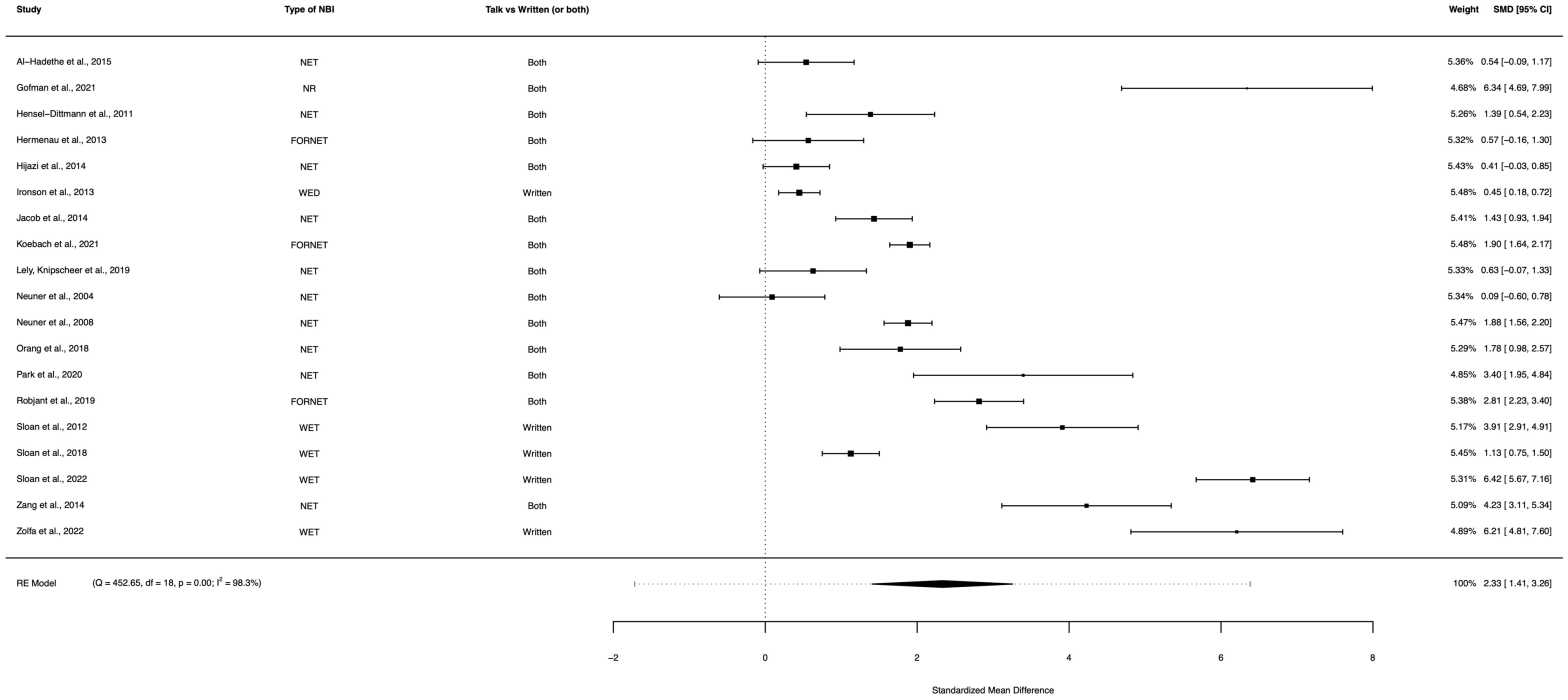
A forest plot indicating the within-group effect size of NBIs from pre-treatment to a 3–9 month follow-up assessment; NET, Narrative Exposure Therapy; NR, Narrative reconstruction; FORNET, Forensic offender rehabilitation narrative exposure therapy; WED, Written emotional disclosure; WET, Written exposure therapy.

A within-group meta-analysis of NBIs (19 studies) from pre-treatment to a 3–9 month follow-up assessment indicated that the effect of NBIs for PTSD symptom reduction was sustained and yielded a greater reduction in symptoms than the initial follow-up. The calculated standardized mean difference yielded a statistically significant pooled effect size of 2.33 (95% CI: 1.41–3.26, *p* < 0.0001; Figure 8). The total amount of heterogeneity in the studies, or tau^2^, was estimated to be 4.05 (*SE* = 1.41). The *I*^2^ statistic was calculated to be 98.25%, indicating a high degree of between study heterogeneity. The *H*^2^ statistic, which quantifies the ratio of total variability to sampling variability, was 57.25. The test for heterogeneity was highly significant [*Q*(*df* = 18) = 452.65, *p* < 0.0001].

### NBI between-group analysis (active controls)

**Figure 9:**
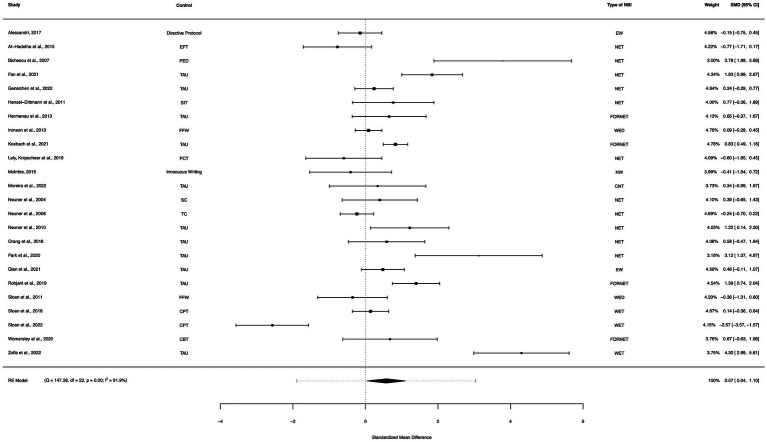
A forest plot indicating the between-group effect size of NBIs compared to active controls from pre- to post-treatment; NET, Narrative Exposure Therapy; EW, Expressive writing; NR, Narrative reconstruction; RTM, Reconsolidation of traumatic memories; FORNET, Forensic offender rehabilitation narrative exposure therapy; WED, Written emotional disclosure; NW, Narrative writing; CNT, Cognitive narrative therapy; WET, Written exposure therapy; EFT, Emotional freedom technique; PED, Psychoeducation; TAU, Treatment as usual; SIT, Stress inoculation training; FFW, Fact-focused writing; PCT, Present centered therapy; SC, Supportive counselling; TC, Trauma counselling; CPT, Cognitive processing therapy.

A between-group meta-analysis of NBIs versus active controls (24 studies) from pre- to post-treatment (subtracting the pooled effect size of active controls from NBIs) indicated that NBIs were more effective in the reduction of PTSD symptoms than active controls. The subtracted calculated standardized mean difference yielded a statistically significant difference in effect size of 0.57 (95% CI: 0.04–1.10, *p* = 0.035; Figure 9). The total amount of heterogeneity in the studies, or tau^2^, was estimated to be 1.50 (*SE* = 0.52). The *I*^2^ statistic was calculated to be 91.92%, indicating a high degree of between study heterogeneity. The *H*^2^ statistic, which quantifies the ratio of total variability to sampling variability, was 12.37. The test for heterogeneity was highly significant [*Q*(*df* = 23) = 147.36, *p* < 0.0001].

### NBI between-group analysis (waitlist controls)

**Figure 10:**
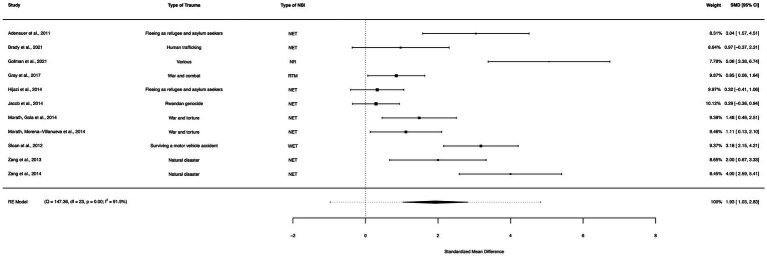
A forest plot indicating the between-group effect size of NBIs compared to waitlist controls from pre- to post-treatment; NET, Narrative Exposure Therapy; NR, Narrative reconstruction; RTM, Reconsolidation of traumatic memories; WET, Written exposure therapy.

A between-group meta-analysis of NBIs versus waitlist controls (11 studies) from pre- to post-treatment (subtracting the pooled effect size of waitlist controls from NBIs) indicated that NBIs were effective in the reduction of PTSD symptoms. The subtracted calculated standardized mean difference yielded a statistically significant difference in effect size of 1.93 (95% CI: 1.03–2.83, *p* < 0.0001; Figure 10). The total amount of heterogeneity in the studies, or tau^2^, was estimated to be 1.97 (*SE* = 1.03). The *I^2^* statistic was calculated to be 88.16%, indicating a high degree of between study heterogeneity. The *H*^2^ statistic, which quantifies the ratio of total variability to sampling variability, was 8.45. The test for heterogeneity was highly significant [*Q*(*df* = 10) = 70.46, *p* < 0.0001].

### Net-only between group analysis (active controls)

**Figure 11:**
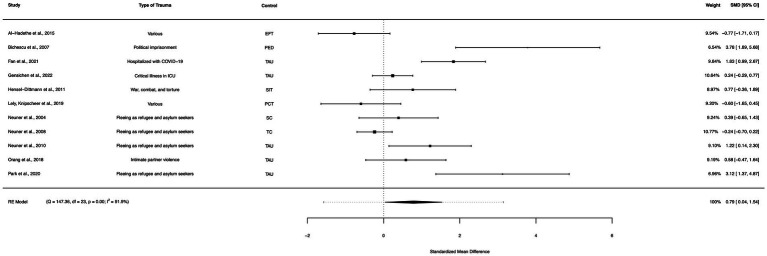
A forest plot indicating the between-group effect size of NET compared to active controls from pre- to post-treatment; NET, Narrative Exposure Therapy; EFT, Emotional freedom technique; PED, Psychoeducation; TAU, Treatment as usual; SIT, Stress inoculation training; FFW, Fact-focused writing; PCT, Present centered therapy; SC, Supportive counselling; TC, Trauma counselling.

A between-group meta-analysis of NET versus active controls (11 studies) from pre- to post-treatment (subtracting the pooled effect size of active controls from NET) indicated that NET was more effective in the reduction of PTSD symptoms than active controls. The subtracted calculated standardized mean difference yielded a statistically significant difference in effect size of 0.79 (95% CI: 0.04–1.54, *p* = 0.039; Figure 11). The total amount of heterogeneity in the studies, or tau^2^, was estimated to be 1.30 (*SE* = 0.71). The *I*^2^ statistic was calculated to be 86.78%, indicating a high degree of between study heterogeneity. The *H*^2^ statistic, which quantifies the ratio of total variability to sampling variability, was 7.56. The test for heterogeneity was highly significant [*Q*(*df* = 10) = 52.44, *p* < 0.0001].

### Net-only between group analysis (waitlist controls)

**Figure 12:**
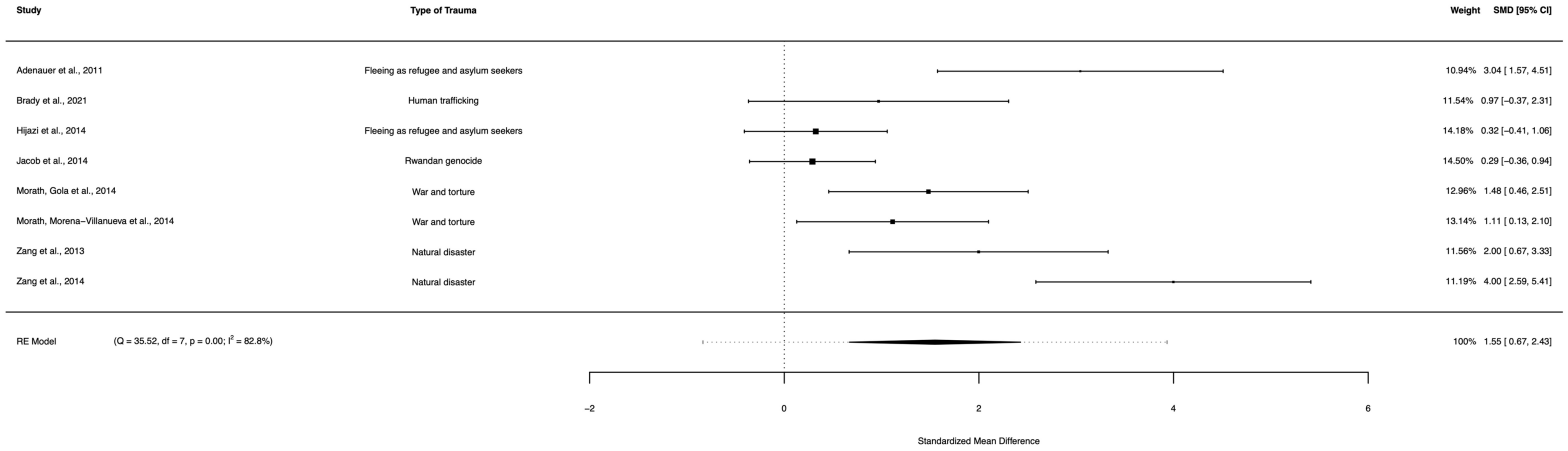
A forest plot indicating the between-group effect size of NET compared to waitlist controls from pre- to post-treatment.

A between-group meta-analysis of NET versus waitlist controls (8 studies) from pre- to post-treatment (subtracting the pooled effect size of waitlist controls from NET) indicated that NET was effective in the reduction of PTSD symptoms. The subtracted calculated standardized mean difference yielded a statistically significant difference in effect size of 1.55 (95% CI: 0.67–2.43, *p* = 0.001; Figure 12). The total amount of heterogeneity in the studies, or tau^2^, was estimated to be 1.28 (*SE* = 0.86). The *I*^2^ statistic was calculated to be 82.78%, indicating a high degree of between study heterogeneity. The *H*^2^ statistic, which quantifies the ratio of total variability to sampling variability, was 5.81. The test for heterogeneity was highly significant [*Q*(*df* = 7) = 35.517, *p* < 0.0001]. A meta-analysis of NET/FORNET versus waitlist controls was not carried out, as there were no studies directly comparing FORNET with a waitlist condition.

### Net/FORNET between group analysis (active controls)

**Figure 13:**
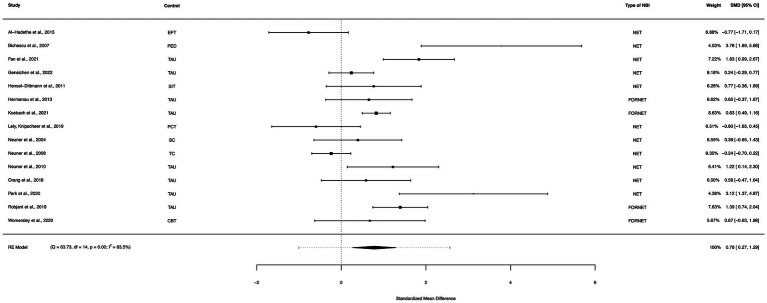
A forest plot indicating the between-group effect size of NET/FORNET compared to active controls from pre- to post-treatment; NET, Narrative Exposure Therapy; FORNET, Forensic offender rehabilitation narrative exposure therapy; EFT, Emotional freedom technique; PED, Psychoeducation; TAU, Treatment as usual; SIT, Stress inoculation training; PCT, Present centered therapy; SC, Supportive counselling; TC, Trauma counselling.

A between-group meta-analysis of NET/FORNET versus active controls (15 studies) from pre- to post-treatment (subtracting the pooled effect size of active controls from NET/FORNET) indicated that NET/FORNET was more effective in the reduction of PTSD symptoms than active controls. The subtracted calculated standardized mean difference yielded a statistically significant difference in effect size of 0.78 (95% CI: 0.27–1.29, *p* = 0.003; Figure 13). The total amount of heterogeneity in the studies, or tau^2^, was estimated to be 0.76 (*SE* = 0.38). The *I^2^* statistic was calculated to be 83.48%, indicating a high degree of between study heterogeneity. The *H*^2^ statistic, which quantifies the ratio of total variability to sampling variability, was 6.05. The test for heterogeneity was highly significant [*Q*(*df* = 14) = 63.73, *p* < 0.0001].

### Small study and publication Bias

**Figure 14:**
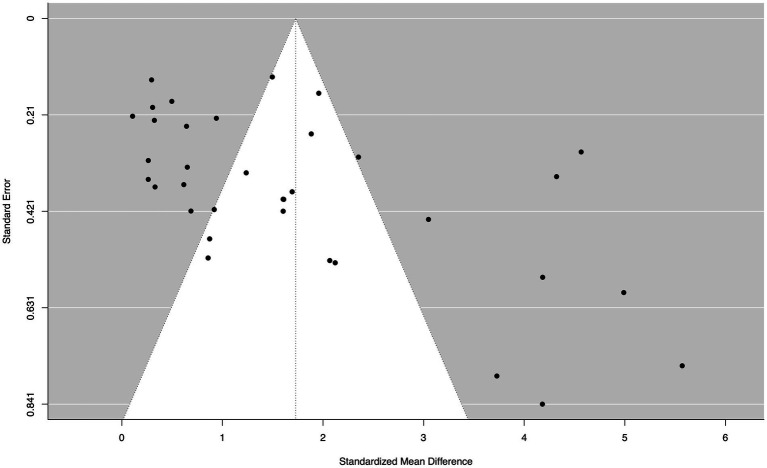
A funnel plot demonstrating significant asymmetry, based on the within-group NBI random effects model from pre- to post-treatment.

**Figure 15:**
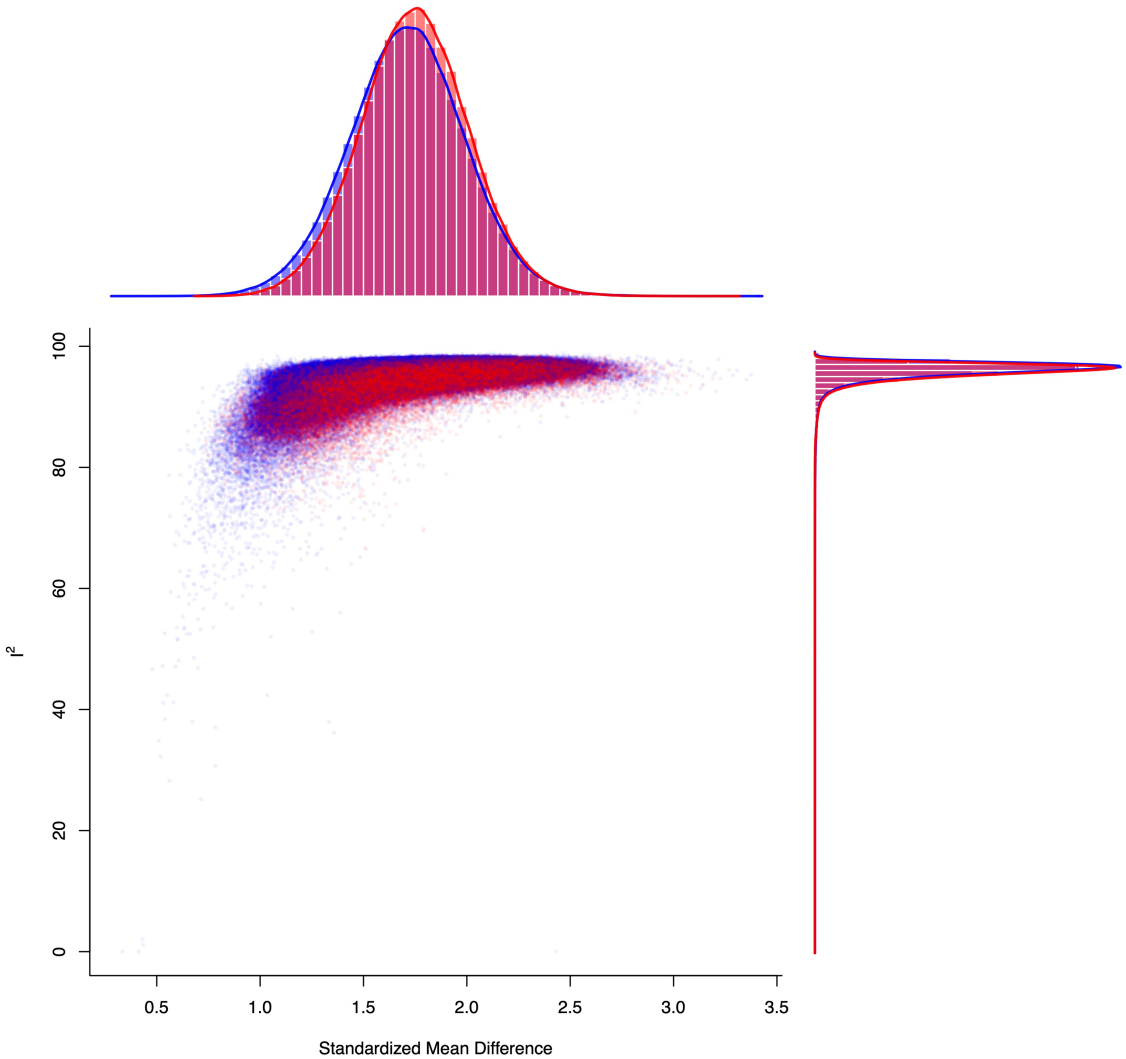
A GOSH plot analysis, fitted to 1e+06 models (based on random subsets) using the NBI within-group random effects model, to display the *I*^2^ statistic against the standardized mean difference. This model suggests a unimodal distribution in both the pooled estimate and the *I*^2^ statistic. Heterogeneity remained significant, regardless of study omission (the points for subsets that include the most significant potential outlier study #9, Gofman et al., 2021—identified via a leave-one-out analysis—are shown in red, whereas the rest are shown in blue).

A funnel plot for the within-group NBI random effects model at the initial post-treatment assessment indicated significant asymmetry (Figure 14). Egger’s regression test for funnel plot asymmetry for the mixed-effects meta-regression model confirmed that there is significant evidence of publication bias (*z* = 4.29, *p* < 0.0001). The limit estimate for the effect size when the standard error approaches 0 was −0.27 (95% CI: −1.25, 0.72). A rank correlation test for funnel plot asymmetry also indicated that there is significant evidence of publication bias (*p* = 0.001). The magnitude of the correlation between effect size and standard error was high (Kendall’s tau = 0.39), indicating that the bias may be affecting the results of the meta-analysis. However, the results from the Vevea and Hedges (1995) weight-function model indicate that there is a significant amount of heterogeneity in the data, even after taking small study and publication bias into account. The likelihood ratio test showed that the adjusted model was not statistically different from the unadjusted model (*df* = 0.41, *p* = 0.522). These findings were confirmed by using the trim-and-fill method, which did not alter the results of our analysis. A GOSH plot was then created, fitted to 1e+06 models (based on random subsets) using the NBI within-group random effects model, to display the *I*^2^ statistic against the standardized mean difference, which indicated a normal distribution of effect sizes, but a high degree of heterogeneity in the dataset (Figure 15).

## Discussion

### Efficacy of NBIs in treating PTSD

This meta-analysis aimed to assess the efficacy of narrative-based interventions (NBIs) in the treatment of post-traumatic stress disorder (PTSD). The overall findings of this analysis suggest that NBIs are an effective treatment for PTSD. The within-group effect size of NBIs indicated a statistically significant reduction in PTSD symptoms, with studies that reported a 3–9 month follow-up assessment after completing an NBI yielding a further statistically significant reduction in symptoms. Although more research is needed to evaluate the sustained effect of NBIs, particularly longitudinal follow-up assessments at one-year post-treatment and beyond, the increase in effect size between the initial post-treatment assessment and 3–9 month follow-up assessment in the present study suggests that NBIs continue to elicit a sustained effect in the months following treatment cessation. However, these results should be interpreted with caution due to the smaller sample size of the subset of studies (19 studies; 1,581 participants) reporting a 3–9 month follow-up assessment compared to the sample size of the initial post-treatment meta-analysis (35 studies; 2,596 participants), therefore increasing risk of bias. Notwithstanding, the overall results of this analysis suggest that NBIs are a viable treatment option for PTSD, effectively producing a substantial, sustained reduction of symptoms. Additionally, all included NBI subgroups [overall NBIs, NET-only, NET/FORNET, and non-(NET/FORNET)] yielded a statistically significant larger effect size than active controls, as well as waitlist controls. This suggests that NBIs may outperform non-NBIs commonly used in clinical settings, most likely due to differences in the treatment protocol between NBIs and non-NBIs, although controlled comparisons are needed to investigate this further. Notably, there was significant evidence of small study and publication bias in the data, but a weight-function model and trim-and-fill method both indicated that this bias was not influencing the results and rather, was most likely due to a high degree of heterogeneity in the data. However, the significant heterogeneity results in our analysis combined with consistency of effect size across unadjusted and adjusted effects models (correcting for small study and publication bias) lends support to the hypothesis that NBIs (as defined in Table 2) are effective in reducing PTSD symptoms.

It may therefore be helpful to clarify the differences between NBIs and non-NBIs to understand why NBIs elicit a stronger effect than other treatment protocols. For example, Narrative Reconstruction (NR) and Narrative Exposure Therapy (NET), both included in this analysis, have distinct similarities that promote a reduction in PTSD symptoms. Both of these NBIs share similar structure and protocol, facilitating a process that reauthors the patient’s self-narrative: re-evaluating and reframing past memories, beliefs, and future aspirations within the patient’s life narrative timeline to form a more positive story. Similarly, Written Exposure Therapy (WET) and other types of emotional writing protocols included in this analysis help the patient work through the narrative aspects of their traumatic experience to make sense of incongruent information (Thompson-Hollands et al., 2019). These narrative-aspects can be juxtaposed with interventions that failed to meet our definition of NBIs, such as Cognitive Processing Therapy (CPT) or Cognitive Behavioral Therapy (CBT), which are notably more directive in nature and limited to challenging existing beliefs (e.g., stuck points in CPT; cognitive distortions or negative thinking in CBT; Resick, 1992; Resick and Schnicke, 1992, 1993; Johnson and Ceroni, 2020), void of explicitly helping the patient to restructure their inner-monologue into a more positive story through a meaning-making process. As a point of comparison, Asmundson et al. (2018) conducted a robust meta-analysis of the efficacy of CPT in PTSD symptom reduction, which showed a positive effect (mean Hedges’ *g =* 1.24), albeit smaller than the effect of NBIs presented in this study. A similar finding was reported for CBT in a meta-analysis conducted by Thielemann et al. (2022), which showed an even smaller effect (mean Hedges’ *g* = 1.14) than CPT, however this analysis notably included children and adolescents. In terms of treatment protocols, it is also helpful to note that NBIs typically require fewer sessions than other established treatments (e.g., NET requires 10 sessions, Schauer et al., 2011; CBT requires 12 sessions, Ehlers, 2013; CPT requires more than 12 sessions, Resick et al., 2017), which may provide more utility in clinical settings. Additionally, included studies from this analysis showed that NBIs outperformed active controls, in which Womersley et al. (2020) used CBT (mean difference in Hedges’ *g =* 0.67) and Sloan et al. (2018) used CPT (mean difference in Hedges’ *g =* 0.67). Another included study, Sloan et al. (2022), used CPT as an active control, but showed a stronger effect for CPT than WET (mean difference in Hedges’ *g =* −2.57). However, this was most likely due to the substantial difference in treatment times between groups in the study design (total treatment times: WET = 240 min; CPT = 720 min), which will have significantly influenced the results.

NBIs utilize strategies that non-NBIs may also implement, such as attending to senses, thoughts, beliefs, and emotions associated with traumatic memories. However, NBIs also seem to facilitate a process of integrating the traumatic memory into the patient’s autobiographical story in a meaningful way (Gofman et al., 2022), although the precise details of this meaning-making process remain unclarified. Moreover, many psychotherapeutic approaches for PTSD focus on fear extinction (e.g., PE or EMDR; Foa and Kozak, 1986; Shapiro and Forrest, 2001), but as Gofman et al. (2022) noted, many cases of PTSD are maintained due to other feelings, such as shame, guilt, and anger—not fear, which NBIs may be better suited to resolve. Importantly, interventions that only focus on fear extinction, such as PE or EMDR, do not appear to explicitly prompt the patient to engage in any type of meaning-making process. Additionally, it is plausible that NBIs are better equipped to address maladaptive future simulations compared to non-NBIs, although more research is needed to substantiate this theory in PTSD etiology.

It is worth noting that there may be other interventions for PTSD that meet our definition of NBI, but were not included in this analysis, just as there may be effective aspects in non-NBI treatment protocols that address the same maladaptive AM distortions. Other interventions for PTSD, such as Imagery Rescripting (IR), could arguably meet the definition for NBIs outlined in Table 2 (Arntz, 2012). IR aims to rewrite traumatic memories to form a more positive perspective of the past, a process which may be facilitated by the AM recovery mechanisms targeted in this analysis. The protocol in IR involves a process of reframing or rescripting the traumatic memory, which helps decrease the emotional salience associated with the memory and, consequently, elicits a reduction in PTSD symptoms. However, the rescripted memory in IR does not need to be based in reality; rather, the explicit goal is to imagine a different outcome of the experience or to alter specific details of the memory to make it as non-traumatic as possible. For example, a 2022 case study detailed this process with a female patient who had been raped and developed PTSD (Lechner-Meichsner et al., 2022). Through IR, the patient was able to reimagine her traumatic experience with alternative outcomes, such as escaping to a quiet and safe place or confronting her rapists and regaining control of the situation. This imaginative exercise reduced the patient’s feelings of contamination, resulting in an overall decrease of 7 points on her CAPS-5 score, bringing her total score below the clinical threshold. However, it remains unclear whether or not IR explicitly helps the patient to form a more positive story by meaningfully integrating the traumatic memory into the patient’s life narrative, or if this process primarily constitutes a more imaginative form of exposure therapy, which is the reason for its exclusion from the present analysis. Importantly, there may also be variability in how clinicians administer various interventions that elicit a positive effect by slight deviations from the protocol. For example, some clinicians may still facilitate a meaning-making process with their patient while engaged in a non-NBI treatment, even though that process is not explicitly outlined as part of the protocol. Given the challenging nature of controlling for this variability, anecdotally observed in clinical settings, it is essential to elucidate the AM mechanisms implicated in PTSD onset and recovery to understand precisely which aspects of various treatment protocols have the strongest impact on symptom reduction and why.

### Traumatic memory processing

Contrary to the typical cognitive processing that occurs in everyday settings, Ehlers and Clark (2000) argued that traumatic experiences are more likely to be processed in a “data-driven” (bottom-up; perceptual) manner, rather than a “conceptual” (top-down; episodic) manner, thereby resulting in fragmented memories characterized by perceptual and sensorial details (also see Roediger, 1990). In this theory, “conceptual processing” refers to one’s ability to understand the meaning of a situation and organize it coherently with previous AM, whereas “data-driven processing” refers to a type of cognitive processing that simply gathers sensory-based information, without the conceptual organization. This line of reasoning, along with other similar theories, is often used to explain why neutral stimuli can trigger an autonomic retrieval of perceptual memories, such as flashbacks or a sensorial experience of reliving the event, without being able to recall episodic details in a manner consistent with everyday AM functioning (Brewin, 2014). Importantly, as noted by Berntsen and Nielsen (2021), involuntary traumatic memories are no more veridical than ordinary AM and there is no evidence suggesting that involuntary perceptual memories contain unprocessed or inaccessible episodic information. Although perceptually vivid, they are still a (re)constructive act in response to environmental cues. Likewise, longitudinal studies have shown that traumatic memories are not static and like ordinary AM, change substantially over time. For instance, Dekel and Bonanno (2013) performed a cross-sectional study to assess changes in traumatic memories in individuals that were inside the World Trade Center during the September 11th terrorist attack. Their findings indicated that only one-third of the initial context of AM from that day remained constant over time and that narrative length also shortened. A similar line of evidence suggests that flashbulb memories for traumatic public events are more durable, but are still nevertheless subject to change, which Talarico and Rubin (2003) observed may actually be a dissociation between belief in accuracy and actual consistency. In non-traumatic circumstances, this flexibility of AM most likely allows the generation of different future scenarios to inform the most appropriate course of action in the present, drawing upon information from the past stored in AM. However, during the encoding and processing of traumatic memories, this dynamic integration of past memory into future simulation is clearly disrupted—resulting in a lack of coherence to the patient’s internal storytelling.

This cognitive processing theory, proposed by Ehlers and Clark (2000), is also supported by neurobiological evidence, as the association between the amygdala and the formation of highly emotional memories is well-established, particularly memories encoded during a fear-provoking experience (Fanselow and Gale, 2003; Rauch et al., 2003; McGaugh, 2004; Phelps and LeDoux, 2005; Sigurdsson et al., 2007; Kim et al., 2011; Paré and Duvarci, 2012). Research suggests that during a traumatic experience, the amygdala may become hyperfunctional as a protective mechanism due to an expectancy violation, preparing the organism for potential future experiences of a similar threat-level (for a review, see Diamond and Zoladz, 2016). It is probable that intrusive and unwanted memories (flashbacks) in PTSD can be attributed to the reactivation of the amygdala provoked by external or mentally induced triggers, which can lead to the spontaneous recall of these memories (Marks et al., 2018). Yet, it remains unclear whether the same process facilitates intrusive future simulations (flashforwards; see Table 1). At the same time, PTSD causes a decrease in activity in parts of the brain that are responsible for higher-level control, such as the medial prefrontal cortex (Shin et al., 2006). As Koenigs and Grafman (2009) observed, individuals with PTSD demonstrate decreased functional connectivity between the medial prefrontal cortex and hippocampus when compared to healthy or traumatized asymptomatic controls, which is most likely the primary cause of impairment in top-down, future-oriented control of AM recall. Despite the impaired functional connectivity between prefrontal and temporal lobes that has been observed in PTSD, the high degree of heterogeneity in psychological responses to trauma may create variability in this dynamic, making it difficult to accurately determine the precise interaction between these two brain areas (Stevens et al., 2018; also see Simons and Spiers, 2003). Although the neurobiological evidence supports cognitive theories on how traumatic memories are initially encoded, it remains unclear what mechanisms support the narrative meaning-making process, highlighted in Figure 2, warranting further investigation. It is likely that this process is dependent on interactions between the prefrontal cortex, hippocampus, and the amygdala, with the ventromedial prefrontal cortex potentially responsible for top-down information integration and the dorsolateral and medial prefrontal cortices responsible for regulating emotional reactions to cues and providing top-down control over the amygdala (Garrett et al., 2019; Harnett et al., 2020; Kredlow et al., 2021). Notably, Lyoo et al. (2011) observed that increased cortical thickness in the dorsolateral prefrontal cortex was associated with better psychological recovery from trauma, providing further support for this theory. It is also clear that the mechanism of fear extinction in exposure therapies involves prefrontal inhibition of the amygdala to reduce the intensity of threatening memories (Dunsmoor et al., 2015). Nevertheless, further research is needed to understand the roles of these prefrontal cortices in various AM phenomena during PTSD onset and recovery, particularly in identifying which brain regions are implicated in the process of conceptually integrating incongruent autobiographical information into an individual’s life story.

This line of neurobiological evidence also helps to provide critical context for one study included in the present analysis that merits discussion. Adenauer et al. (2011), indicated via magnetoencephalography (MEG) evaluations of neuromagnetic oscillations in the brain (known as steady-state visual evoked fields, or ssVEF) that NET resulted in an increase of cortical top-down attentional regulation when participants were shown aversive pictures. This is highly relevant to the current topic of resolving maladaptive AM phenomena in PTSD, as visuospatial imagination is not only critical to the process of retrieving information from AM, but it is also vitally pertinent to how individuals process fear-provoking and intrusive memories (Lang, 1977; Lin-Stephens, 2020). This neuroimaging evidence suggests that NBIs, specifically NET in this instance, may help improve temporal attentional control in AM recall, thereby minimizing distressing AM distortions or intrusive traumatic cognitions. Moreover, this evidence points to the possibility that reconciling incongruent traumatic memories with the patient’s overall life narrative (outlined in Figure 2) may improve the temporal control of retrospective and prospective AM by accounting for expectancy violations. To explore this hypothesis of attentional control in the patient’s ability to mental time travel, it would be beneficial to elucidate whether or not future temporal dimensions have the same maladaptive distributions in PTSD onset and whether or not they are corrected in the recovery process.

### The adaptive benefits of narrative structuring in AM

There are many proposed theories in the literature on the adaptive benefits for having a psychological response to trauma in the first place, which can be difficult to reconcile. However, it is important to note that trauma responses are healthy responses from healthy individuals to traumatic experiences, and many human fear responses are “pre-programmed” based on previously threatening stimuli throughout human evolution (e.g., visual recognition of objects that resemble snakes; see Van Le et al., 2013, 2016). Given this evidence and the results of this analysis, perhaps the true adaptive benefit of memory is not in how accurate it is, but how *useful* it can be in organizing an individual’s personal past to mentally structure a more positive and safe prospective future. This adaptive benefit is also reflected in the physical health outcomes of patients from included studies of this analysis. It is well-established that PTSD has a positive correlation with various physiological pathologies, such as autoimmune diseases, cancer, cardiovascular diseases, chronic pain, immunological functioning, as well as gastrointestinal issues (Boscarino, 2004; Pace and Heim, 2011; Kolassa et al., 2015). It is therefore helpful to briefly discuss important physiological findings from NBI studies included in this analysis to further highlight their efficacy in PTSD treatment. For example, Neuner et al. (2008) showed that NET reduced the frequency and intensity of coughing, gastrointestinal problems, and fever in patients. More so, Morath et al. (2014a,b) found that NBIs for PTSD (NET) were not only superior to active controls, but that they actually aided in repairing DNA breakage and improving immunological functioning, both of which are often disrupted during PTSD onset. Additionally, Womersley et al. (2020) showed that FORNET was associated with an increase in DNA methylation, which the authors theorized may be a mediator of the beneficial effects in symptom reduction, although further studies are needed to understand this potential mechanism. To further this line of evidence, Wilker et al. (2023) investigated the epigenetic expression of PTSD onset and recovery, particularly examining the methylation at CpG site cg25535999, which was found to be inversely related to PTSD symptoms. Importantly, the data from this study indicate that NET was effective in reducing symptoms and resulted in a significant increase in cg25535999 methylation, which the authors argue highlights the importance of glucocorticoids in the recovery process (De Quervain et al., 2019). This line of evidence is supported by research investigating the evolutionary history of PTSD-associated CpG sites, which not only tracks the phylogenetic history of PTSD through its associated CpG dinucleotide, but more so provides insight into the epigenetic potential in humans to respond to traumatic experiences through resilient narratives (Sipahi et al., 2014). Overall, these results raise new questions about the adaptive neurophysiological benefits of the theorized narrative structure of AM more generally and suggests that this narrative structuring or the ability to build meaningful coherence within an individual’s life story may have played an important evolutionary role in processing experiential information while maintaining an optimal biological stasis, particularly in making sense of potentially incongruent, dangerous, and novel information (McAdams, 2019; also see Garcia-Pelegrin et al., 2021).

### The question of narrative incoherence

At first glance, it would make sense that NBIs should specifically help to counter the problem of narrative incoherence in PTSD. However, it is important to note that the issue of narrative incoherence still has many unanswered questions, with evidence both for and against common claims (for a recent review, see Crespo and Fernández-Lansac, 2016). Additionally, there is an ongoing debate in the literature regarding the fragmentation of traumatic memories, fueled by discrepancies in findings from studies involving clinical versus non-clinical samples (McNally, 2022). However, these discrepancies may be further exacerbated by considerable heterogeneity regarding conceptual definitions of cognitive narratives, which structure many facets of an individual’s mental life through the AM system, such as identity, beliefs, social interactions, future goals, perceptions, and the capacity for fictional storytelling (Clayton and Wilkins, 2017; Wilkins and Clayton, 2019; also see Adler, 2012). This heterogeneity makes it difficult to reconcile various measurements of cognitive narratives and potential fragmentation, even though the overall study design of narrative-focused research typically follows the same structure. For example, Jaeger et al. (2014) examined how word usage in traumatic memories of individuals with PTSD may indicate narrative structuring, whereas Fitzke et al. (2021) investigated narratives as a meaning-making process implicated in the recovery of PTSD by rating coherence, significance, and sense of purpose. It is evident that these studies are not defining or measuring narratives consistently and future research should aim to address this discrepancy.

Assuming that narrative incoherence is an observable phenomenon associated with PTSD, it still remains unclear whether this phenomenon is a consequence of PTSD onset, a contributory factor to its etiology, or both. For example, a related line of evidence from Sutin et al. (2021) found that individuals who report feeling a higher “sense of purpose” in life correlated strongly with also having richer and more detailed AM recall. Yet, it is also well-documented that feeling a strong sense of purpose predicts better emotional recovery from traumatic events and fewer symptoms of PTSD (Schaefer et al., 2013). It is therefore possible that traumatic events could disrupt this sense of purpose, or life narrative coherence, which may create deficits in AM. However, it could also be reasoned (and has been, see Hirsh et al., 2013), that coherent personal narratives, from which individuals derive a sense of purpose or meaning in life, may be evidence of the highest level of cognitive integration, particularly in processing traumatic experiences: structuring future expectations and minimizing predictive errors. Evidence of this phenomenon can be observed in a recent case study using a narrative approach as intervention for a veteran with PTSD, which showed that autobiographical writing by the individual of their experience pre-, mid-, and post-trauma helped to turn highly distressing memories into meaningful experience, thereby also significantly reducing symptoms and increasing positive future-thinking (Muijnck, 2022). A related line of evidence seems to support this theory, indicating that life narratives significantly influence resilience factors and outcomes from potentially distressing events (Ramasubramanian et al., 2022). As such, stronger indicators of lexical markers in memory recall, such as meaning-making and integration into one’s life narrative, also typically predict fewer symptoms and better outcomes after a stressful event (Park, 2010). For instance, Follmer Greenhoot et al. (2013), obtained a “narrative structure score” by analyzing the context, meaning-making, and chronology of traumatic memories in adults with abusive histories and found that narrative aspects, such as positive meaning-making of the event, were the best predictors of psychological adjustment for PTSD. Inversely, Kleim et al. (2018) observed that orally collected narrative transcripts about a traumatic experience with less cognitive processing words (indicating less AM integration or elaboration) predicted greater PTSD symptom severity 6 months after trauma-exposure, which further highlights the disturbance in narrative processing incurred during PTSD onset and the importance of establishing coherence in the recovery process.

It is also well-established that outcomes for PTSD vary depending on numerous factors, of which the type of traumatic experience and the method of initial disclosure are particularly noteworthy. For example, Gray and Lombardo (2001) found that higher levels of anxiety during the initial disclosure created more fragmented narratives in memories, which negatively influenced patient outcomes. Lindblom and Gray (2010) found that higher degrees of betrayal in the traumatic event itself were also associated with more fragmented narratives in memories and likewise, poorer outcomes. Interestingly, they also found that victims of perpetuated trauma had more overall symptoms and a higher degree of fragmentation than victims of accidental trauma, suggesting that the nature of the traumatic event itself and the actual content of the memory significantly influences the development and abatement of symptoms. These findings help to explain why the within-group sub-analysis of NET/FORNET had a smaller effect than the NET-only group, since individuals undergoing the FORNET intervention are typically criminal offenders and therefore processing a different trauma narrative than victims. There is also a high degree of potential heterogeneity with regards to the “trauma load” in participants included in this analysis, which refers to the build-up of previous traumas throughout the lifetime prior to the onset of clinically diagnosable PTSD (Neuner et al., 2004). However, Schneider et al. (2020) showed that the effect of NET on PTSD symptom reduction is independent of trauma load, but that individuals with a higher exposure to past traumatic events may require more sessions or additional treatment time in order to have the same total symptom reduction as other participants.

### Contextualizing results within the literature

Overall, our findings are consistent with other meta-analyses on specific NBIs, such as a recent meta-analysis by Wei and Chen (2021), which indicated that NET had a moderate effect for PTSD symptom reduction compared to active controls and ultimately supported the efficacy of NET for loss of diagnosis. Another meta-analysis, Lely et al. (2019b) similarly showed that NET resulted in sustained symptom abatement in PTSD. In the present analysis, non-(NET/FORNET) NBIs yielded a more significant effect size than NET/FORNET interventions, but this data should be interpreted with caution as there is a high degree of heterogeneity among non-(NET/FORNET) NBIs. A controlled comparison between various NBIs, including NET/FORNET and other NBIs previously analyzed, is warranted to further understand the efficacy of these interventions and to investigate how the meaning-making process precisely occurs after a traumatic experience. A related meta-analysis assessing all types of exposure therapies as intervention for PTSD, including NET and WET, demonstrates the overall efficacy of the premise behind exposure (e.g., confronting fear-evoking memories; McLean et al., 2022), but it remains unclear whether this type of exposure deals with mentally evoked fear-stimuli from both retrospective and prospective memories (e.g., the capacity for future simulations), which is a question that future research should address. Finally, the results of this analysis contribute to a growing body of evidence (e.g., Cohn-Sheehy et al., 2021a,b) suggesting that a narrative structure plays a crucial role within AM, requiring further investigation.

### Limitations

There are few limitations in this analysis that require attention. First, as with many studies on the efficacy of PTSD interventions, there is a high risk that patients who agree to participate are typically those who are less avoidant and therefore also more likely to be successful with treatment. It has also been observed, as was the case in Dekel and Bonanno’s (2013) study on survivors from the September 11th terrorist attack, that patients with more severe symptoms are less likely to seek participation in PTSD studies and to relocate instead. Notably, recruitment for studies investigating traumatic memories in individuals with PTSD is a difficult task and can often result in biased data, as there is a high degree of heterogeneity in types of traumatic experiences that can produce an onset of PTSD and many disagreements in the literature on how to define trauma precisely (May and Wisco, 2016).

There is also a limitation in comparing the administration of treatment protocols, particularly in relation to the interaction of timescales among included studies. The variability in treatment protocols, stemming from the inclusion of both manualized and self-guided protocols, contributed to a high degree heterogeneity in pre- and post-treatment assessment times. In this analysis, the potential time interaction from pre- to post-treatment ranges from 2 weeks to 6 months. Despite that, the follow-up data included in our analysis, of which we selected the latest available assessment reported within the 3–9 month post-treatment follow-up range, nonetheless indicated a sustained, statistically significant effect size. However, the dose–response relationship in PTSD remains unclear and is often obscured by symptom overreporting, as well as the interaction of complex trauma loads (Merckelbach et al., 2014).

Finally, conceptual, definitional, and methodological variability of cognitive narratives require further elucidation and reconciliation. Although our analysis is largely based on the *a priori* assumption that narrative integration is implicit in the recovery process from PTSD, our results indicate that NBIs yield a reduction in PTSD symptoms. However, it is evident throughout the literature cited in this analysis that many researchers hold different concepts or definitions of cognitive narratives, which raises many questions and concerns on the validity and comparability of this line of research writ large. Future research should therefore aim to understand the phenomenology of cognitive narratives on a more fundamental level, as well as how and why they are involved in the process of retrospective and prospective memory, in both local and global resolutions.

### Future directions

Due to PTSD often leaving people feeling helpless or wary about the future, it would make sense if this diagnosis were more so related to impairments in the capacity for prospective future simulations and not merely retrospective AM recall; however so far empirical evidence supporting this idea remains extremely sparse. For example, survivors of the Chernobyl nuclear disaster reported impairments in their ability to simulate the future, referred to as flashforwards, which included intrusive mental simulations of future events in which they would develop major health complications due to radiation exposure incurred during the traumatic event (Loganovsky and Zdanevich, 2013). This process dramatically altered the individual’s mental rehearsals for their prospective future and most likely contributed substantially to the maintenance of PTSD symptoms. This line of research is also supported by preliminary evidence from Steil et al. (2022), in which findings indicated that individuals with PTSD from childhood abuse had a significantly higher degree of intrusive negative mental imagery compared to healthy subjects – although these results should be interpreted with caution due to several potentially confounding variables (e.g., education levels, immigration status, and general intelligence). However, it is also well-established that individuals with clinical depression and co-occurring suicidal ideations also experience intrusive mental imagery, which could be considered flashforwards, in which they exhibit increased mental images of self-harm, their own death, or the death of a loved one (Holmes et al., 2007; Ng et al., 2016; Schultebraucks et al., 2020). As Lely et al. (2019b) observed, age is a predictor of outcome from PTSD, with those in advanced age typically having better recovery results, which can potentially be explained by theories on life narratives (e.g., those with advanced age have stronger and more elaborated past narratives to draw from to make sense of their experience; also see Rizvi et al., 2009). However, it could also be reasoned that older individuals have a smaller scope of potential future simulations and less overall uncertainty about their prospective futures, which could therefore provide an easier path to recovery, especially if an impairment in capacity for future simulation is a primary mechanism implicated in PTSD. From an evolutionary perspective, this theory also makes sense, as it has been observed that all organisms capable of long-term memory seem to be oriented towards the future, specifically towards solving future problems and avoiding potentially dangerous future scenarios (Klein et al., 2010). Notably, the cognitive ability to simulate the future has also been associated with various aspects like social emotions (e.g., regret from unmet expectations), episodic memory rehearsal capabilities, and problem-solving strategies (Schacter, 2019). If an impairment in the capacity for future simulation is also a primary symptom of PTSD, it could shed light on why more fact-focused interventions, such as stuck points and Socratic questioning—as used in CPT, may also address the same maladaptive AM distortions by helping to properly orient the individual towards the future by minimizing predictive errors, consequently improving their sense of narrative identity. Therefore, future research should aim to elucidate whether the problem of coherence involves the capability for both retrospective and prospective memory by investigating the full range of one’s capacity of mental time traveling within individuals diagnosed with PTSD compared to healthy controls (including the potential for impairment in Theory of Mind and in processing fictional information). It would also be helpful for future research to identify the neural correlates that underpin the process of reappraisal and assimilation of incongruent autobiographical information into the individual’s prior ongoing mental narrative to further isolate how and why this process occurs. This will help identify where the primary memory impairments form during PTSD onset and subsequent downstream effects that maintain symptoms, which will be beneficial in understanding the structure of memory more generally. This crossover between cognitive psychology, neuroscience, and clinical psychiatry will be beneficial in a number of ways: (1) by investigating the full range of both retrospective memory and prospective future simulations within individuals diagnosed with PTSD to better understand the mechanisms that give rise to symptoms; (2) by using this information to then assess how to improve interventions and treatment protocols to better address these specific mechanisms; (3) by helping to elucidate the question of narrative coherence more clearly within PTSD etiology, which should help to clarify the form, function, and neurobiological mechanisms of narratives within human cognition more generally.

## Conclusion

This meta-analysis provides important insight and definitional structure on the attributes of NBIs, which yielded statistically significant results in the reduction of PTSD symptoms in each sub-analysis reported. This could be because NBIs seem to address maladaptive AM distortions and the disruption to an individual’s life narrative coherence incurred during PTSD onset in ways that non-NBIs do not, most likely through a meaning-making process. However, additional studies are needed to more clearly elucidate the AM mechanisms implicated in the etiology of PTSD, specifically whether impairments in future simulations are a primary component of pathogenesis, which should then shed light on why some treatment protocols may be more effective than others. More broadly, a greater comprehension of how AM is implicated in other psychiatric disorders could not only aid in elucidating a greater understanding of AM, but also in developing better manualized intervention protocols to address maladaptive AM symptoms. Overall, this line of research will hopefully contribute significantly to a more clarified narrative model of mind and a deeper understanding of the structure of cognitive narratives in AM.

## Data availability statement

The raw data supporting the conclusions of this article will be made available by the authors, without undue reservation.

## Author contributions

RR performed the literature review, study selection, data extraction, statistical analysis, and wrote the manuscript. MB supervised each of these tasks, provided feedback, and edited the manuscript. NC supervised the overall project, provided feedback, and edited the manuscript. All authors contributed to the article and approved the submitted version.

## Conflict of interest

The authors declare that the research was conducted in the absence of any commercial or financial relationships that could be construed as a potential conflict of interest.

## Publisher’s note

All claims expressed in this article are solely those of the authors and do not necessarily represent those of their affiliated organizations, or those of the publisher, the editors and the reviewers. Any product that may be evaluated in this article, or claim that may be made by its manufacturer, is not guaranteed or endorsed by the publisher.

## Supplementary material

The Supplementary material for this article can be found online at: https://www.frontiersin.org/articles/10.3389/fpsyg.2023.1215225/full#supplementary-material

## References

[ref1] Adenauer H. Catani C. Gola H. Keil J. Ruf M. Schauer M.. (2011). Narrative exposure therapy for PTSD increases top-down processing of aversive stimuli – evidence from a randomized controlled treatment trial. BMC Neurosci. 12:127. doi: 10.1186/1471-2202-12-127 22182346 PMC3258226

[ref2] Adler J. M. (2012). Living into the story: agency and coherence in a longitudinal study of narrative identity development and mental health over the course of psychotherapy. J. Pers. Soc. Psychol. 102, 367–389. doi: 10.1037/a0025289, PMID: 21910554

[ref3] Alessandri F. T. (2017). Testing a brief directive intervention to reduce symptoms associated with trauma. eGrove Available at: https://egrove.olemiss.edu/etd/829/

[ref5] Al-Hadethe A. Hunt N. Al-Qaysi G. Thomas S. (2015). Randomised controlled study comparing two psychological therapies for posttraumatic stress disorder (PTSD): emotional freedom techniques (EFT) vs. Narrative Exposure Therapy (NET). J. Trauma. Stress Disord. Treat. 4:4. doi: 10.4172/2324-8947.1000145

[ref6] American Psychiatric Association. (2013). Diagnostic and statistical manual of mental disorders (5th ed.). Washington, DC: American Psychiatric Association

[ref7] Arntz A. (2012). Imagery rescripting as a therapeutic technique: review of clinical trials, basic studies, and research agenda. J. Exp. Psychopathol. 3, 189–208. doi: 10.5127/jep.024211

[ref9001] Ashby F. G. O’Brien J. B. (2005). Category learning and multiple memory systems. Trends Cogn. Sci. 9, 83–89. doi: 10.1016/j.tics.2004.12.003 15668101

[ref8] Asmundson G. J. Thorisdottir A. S. Roden-Foreman J. W. Baird S. O. Witcraft S. M. Stein A. T.. (2018). A meta-analytic review of cognitive processing therapy for adults with posttraumatic stress disorder. Cogn. Behav. Ther. 48, 1–14. doi: 10.1080/16506073.2018.1522371 30332919

[ref9] Avdi E. Georgaca E. (2007). Narrative research in psychotherapy: a critical review. Br. J. Med. Psychol. 80, 407–419. doi: 10.1348/147608306x158092 17877865

[ref10] Barker T. J. Stone J. C. Sears K. Klugar M. Tufanaru C. Leonardi-Bee J.. (2023). The revised JBI critical appraisal tool for the assessment of risk of bias for randomized controlled trials. JBI Evid. Synth. 21, 494–506. doi: 10.11124/jbies-22-00430 36727247

[ref11] Bartlett F. C. (1932). Remembering: a study in experimental and social psychology. Cambridge: Cambridge University Press.

[ref12] Beierl E. T. Böllinghaus I. Clark D. M. Glucksman E. Ehlers A. (2020). Cognitive paths from trauma to posttraumatic stress disorder: a prospective study of Ehlers and Clark’s model in survivors of assaults or road traffic collisions. Psychol. Med. 50, 2172–2181. doi: 10.1017/S0033291719002253 31507261 PMC7557160

[ref13] Berntsen D. Hall N. M. (2004). The episodic nature of involuntary autobiographical memories. Mem. Cogn. 32, 789–803. doi: 10.3758/bf03195869 15552356

[ref14] Berntsen D. Nielsen N. P. (2021). The reconstructive nature of involuntary autobiographical memories. Memory 30, 31–36. doi: 10.1080/09658211.2021.1872645 33459150

[ref15] Berntsen D. Willert M. Rubin D. C. (2003). Splintered memories or vivid landmarks? Qualities and organization of traumatic memories with and without PTSD. Appl. Cogn. Psychol. 17, 675–693. doi: 10.1002/acp.894

[ref16] Bichescu D. Neuner F. Schauer M. Elbert T. (2007). Narrative exposure therapy for political imprisonment-related chronic posttraumatic stress disorder and depression. Behav. Res. Ther. 45, 2212–2220. doi: 10.1016/j.brat.2006.12.006 17288990

[ref17] Blevins C. A. Weathers F. W. Davis M. T. Witte T. K. Domino J. L. (2015). The posttraumatic stress disorder checklist for DSM-5 (PCL-5): development and initial psychometric evaluation. J. Trauma. Stress. 28, 489–498. doi: 10.1002/jts.22059 26606250

[ref18] Boscarino J. A. (2004). Posttraumatic stress disorder and physical illness: results from clinical and epidemiologic studies. Ann. N. Y. Acad. Sci. 1032, 141–153. doi: 10.1196/annals.1314.011 15677401

[ref19] Brady F. Chisholm A. Walsh E. Ottisova L. Bevilacqua L. Mason C.. (2021). Narrative exposure therapy for survivors of human trafficking: feasibility randomised controlled trial. BJPsych. Open 7:e196. doi: 10.1192/bjo.2021.1029

[ref20] Brewin C. R. (2014). Episodic memory, perceptual memory, and their interaction: foundations for a theory of posttraumatic stress disorder. Psychol. Bull. 140, 69–97. doi: 10.1037/a0033722 23914721

[ref21] Brewin C. R. Dalgleish T. Joseph S. (1996). A dual representation theory of posttraumatic stress disorder. Psychol. Rev. 103, 670–686. doi: 10.1037/0033-295X.103.4.670, PMID: 8888651

[ref22] Briere J. Elliott D. M. (1998). Clinical utility of the impact of event scale: psychometrics in the general population. Assessment 5, 171–180. doi: 10.1177/107319119800500207 9626392

[ref23] Brown A. D. Antonius D. Kramer M. Root J. C. Hirst W. (2010). Trauma centrality and PTSD in veterans returning from Iraq and Afghanistan. J. Trauma. Stress 23, 496–499. doi: 10.1002/jts.20547 20690194

[ref24] Brown A. D. Root J. C. Romano T. A. Chang L. J. Bryant R. A. Hirst W. (2013). Overgeneralized autobiographical memory and future thinking in combat veterans with posttraumatic stress disorder. J. Behav. Ther. Exp. Psychiatry 44, 129–134. doi: 10.1016/j.jbtep.2011.11.004 22200095

[ref9002] Budson A. E. Price B. H. (2003). Memory: clinical disorders. Encycl. Earth Sci. doi: 10.1038/npg.els.0002201

[ref25] Carlson E. B. (2001). Psychometric study of a brief screen for PTSD: assessing the impact of multiple traumatic events. Assessment 8, 431–441. doi: 10.1177/107319110100800408 11785587

[ref26] Catarino A. Küpper C. S. Werner-Seidler A. Dalgleish T. Anderson M. C. (2015). Failing to forget. Psychol. Sci. 26, 604–616. doi: 10.1177/0956797615569889 25847536 PMC4426138

[ref27] Clayton N. Wilkins C. (2017). Memory, mental time travel and the Moustachio quartet. Interface Focus 7:20160112. doi: 10.1098/rsfs.2016.0112 28479980 PMC5413891

[ref28] Cloitre M. Shevlin M. Brewin C. R. Bisson J. I. Roberts N. P. Maercker A.. (2018). The international trauma questionnaire: development of a self-report measure of ICD-11 PTSD and complex PTSD. Acta Psychiatr. Scand. 138, 536–546. doi: 10.1111/acps.12956 30178492

[ref29] Cohn-Sheehy B. I. Delarazan A. I. Reagh Z. M. Crivelli-Decker J. Kim K. Barnett A. J.. (2021a). The hippocampus constructs narrative memories across distant events. Curr. Biol. 31, 4935–4945.e7. doi: 10.1016/j.cub.2021.09.013 34592172 PMC9373723

[ref9003] Cohn-Sheehy B. I. Delarazan A. I. Crivelli-Decker J. Reagh Z. M. Mundada N. S. Yonelinas A. P. (2021b). Narratives bridge the divide between distant events in episodic memory. Mem. Cogn. 50, 478–494. doi: 10.3758/s13421-021-01178-x PMC8546012 33904017

[ref30] Conway M. A. (1990). Autobiographical memory: an introduction. Maidenhead: Open University Press.

[ref31] Conway M. A. (1996). “Autobiographical memory” in Memory. eds. Bjork E. L. Bjork R. A. (London: Academic Press), 165–194.

[ref32] Conway M. A. Pleydell-Pearce C. W. (2000). The construction of autobiographical memories in the self-memory system. Psychol. Rev. 107, 261–288. doi: 10.1037/0033-295x.107.2.261 10789197

[ref33] Conway M. A. Rubin D. C. (1993). “The structure of autobiographical memory” in Theories of memory. eds. Collins A. F. Gathercole S. E. Conway M. A. (Hove, UK: Erlbaum), 103–137.

[ref34] Crespo M. Fernández-Lansac V. (2016). Memory and narrative of traumatic events: a literature review. Psychol. Trauma Theory Res. Pract. Policy 8, 149–156. doi: 10.1037/tra0000041 25915647

[ref35] Davidson J. R. Book S. W. Colket J. T. Tupler L. A. Roth S. David D.. (1997). Assessment of a new self-rating scale for post-traumatic stress disorder. Psychol. Med. 27, 153–160. doi: 10.1017/s0033291796004229 9122295

[ref36] De Quervain D. Wolf O. T. Roozendaal B. (2019). Glucocorticoid-induced enhancement of extinction—from animal models to clinical trials. Psychopharmacology 236, 183–199. doi: 10.1007/s00213-018-5116-0 30610352 PMC6373196

[ref37] Dekel S. Bonanno G. A. (2013). Changes in trauma memory and patterns of posttraumatic stress. Psychol. Trauma Theory Res. Pract. Policy 5, 26–34. doi: 10.1037/a0022750

[ref38] Diamond D. A. Zoladz P. R. (2016). Dysfunctional or hyperfunctional? The amygdala in posttraumatic stress disorder is the bull in the evolutionary China shop. J. Neurosci. Res. 94, 437–444. doi: 10.1002/jnr.23684 26511328

[ref39] Dunsmoor J. E. Niv Y. Daw N. Phelps E. A. (2015). Rethinking extinction. Neuron 88, 47–63. doi: 10.1016/j.neuron.2015.09.028 26447572 PMC4598943

[ref40] Duval S. Tweedie R. (2000). Trim and fill: a simple funnel-plot-based method of testing and adjusting for publication bias in meta-analysis. Biometrics 56, 455–463. doi: 10.1111/j.0006-341x.2000.00455.x 10877304

[ref41] Ehlers A. (2013). “Trauma-focused cognitive behavior therapy for posttraumatic stress disorder and acute stress disorder” in CBT for anxiety disorders: a practitioner book. eds. Simos G. Hofmann S. G. (New York: John Wiley & Sons), 161–189. doi: 10.1002/9781118330043.ch7

[ref42] Ehlers A. Clark D. M. (2000). A cognitive model of posttraumatic stress disorder. Behav. Res. Ther. 38, 319–345. doi: 10.1016/s0005-7967(99)00123-0 10761279

[ref43] Fan Y. Shi Y. Zhang J. Sun D. Wang X. Fu G.. (2021). The effects of narrative exposure therapy on COVID-19 patients with post-traumatic stress symptoms: a randomized controlled trial. J. Affect. Disord. 293, 141–147. doi: 10.1016/j.jad.2021.06.019 34186232 PMC8234566

[ref44] Fanselow M. S. Gale G. D. (2003). The amygdala, fear, and memory. Ann. N. Y. Acad. Sci. 985, 125–134. doi: 10.1111/j.1749-6632.2003.tb07077.x 12724154

[ref45] Fitzke R. E. Marsh D. R. Prince M. A. (2021). A longitudinal investigation of the meaning-making model in midlife adults who have experienced trauma. J. Clin. Psychol. 77, 2878–2893. doi: 10.1002/jclp.23272 34709654

[ref46] Fivush R. Booker J. A. Graci M. E. (2017). Ongoing narrative meaning-making within events and across the life span. Imagin. Cogn. Pers. 37, 127–152. doi: 10.1177/0276236617733824

[ref47] Foa E. B. Kozak M. J. (1986). Emotional processing of fear: exposure to corrective information. Psychol. Bull. 99, 20–35. doi: 10.1037/0033-2909.99.1.20, PMID: 2871574

[ref48] Foa E. B. McLean C. P. Zang Y. Zhong J. Powers M. B. Kauffman B. Y.. (2016a). Psychometric properties of the posttraumatic diagnostic scale for DSM-5 (PDS-5). Psychol. Assess. 28, 1166–1171. doi: 10.1037/pas0000258 26691504

[ref49] Foa E. B. McLean C. P. Zang Y. Zhong J. Rauch S. Porter K.. (2016b). Psychometric properties of the posttraumatic stress disorder symptom scale interview for DSM–5 (PSSI–5). Psychol. Assess. 28, 1159–1165. doi: 10.1037/pas0000259 26691507

[ref50] Follmer Greenhoot A. Sun S. Bunnell S. L. Lindboe K. (2013). Making sense of traumatic memories: memory qualities and psychological symptoms in emerging adults with and without abuse histories. Memory 21, 125–142. doi: 10.1080/09658211.2012.712975 22943468

[ref51] Garcia-Pelegrin E. Wilkins C. Clayton N. S. (2021). The ape that lived to tell the tale. The evolution of the art of storytelling and its relationship to mental time travel and theory of mind. Front. Psychol. 12:755783. doi: 10.3389/fpsyg.2021.755783 34744932 PMC8569916

[ref52] Garrett A. Cohen J. G. Zack S. E. Carrion V. G. Jo B. Blader J. C.. (2019). Longitudinal changes in brain function associated with symptom improvement in youth with PTSD. J. Psychiatr. Res. 114, 161–169. doi: 10.1016/j.jpsychires.2019.04.021 31082658 PMC6633919

[ref53] Gensichen J. Friemel C. Schmidt K. Sanftenberg L. Dohmann J. Reips U. D.. (2022). A primary care-based narrative exposure therapy on patients with post-traumatic stress disorder following intensive care. Am. J. Respir. Crit. Care Med. 205:A5363. doi: 10.1164/ajrccm-conference.2022.205.1_meetingabstracts.a5363

[ref54] Gofman M. Kivity Y. Bar-Kalifa E. Vidan Z. Ohayon I. H. Tuval-Mashiach R.. (2021). Narrative reconstruction as an intervention for posttraumatic stress disorder: a pilot delayed intervention quasi-randomized controlled trial. J. Trauma. Stress. 34, 92–103. doi: 10.1002/jts.22537 32521097

[ref55] Gofman M. Kivity Y. Peri T. (2022). Beyond the conditioned fear model: narrative reconstruction for a woman with posttraumatic stress disorder. J. Psychother. Integr. 32, 210–224. doi: 10.1037/int0000262

[ref56] Gray R. Budden-Potts D. Bourke F. (2017). Reconsolidation of traumatic memories for PTSD: a randomized controlled trial of 74 male veterans. Psychother. Res. 29, 621–639. doi: 10.1080/10503307.2017.1408973 29241423

[ref57] Gray M. J. Lombardo T. W. (2001). Complexity of trauma narratives as an index of fragmented memory in PTSD: a critical analysis. Appl. Cogn. Psychol. 15, S171–S186. doi: 10.1002/acp.840

[ref58] Gwozdziewycz N. Mehl-Madrona L. (2013). Meta-analysis of the use of narrative exposure therapy for the effects of trauma among refugee populations. Perm. J. 17, 70–76. doi: 10.7812/TPP/12-058 23596375 PMC3627789

[ref59] Halligan S. L. Michael T. Clark D. M. Ehlers A. (2003). Posttraumatic stress disorder following assault: the role of cognitive processing, trauma memory, and appraisals. J. Consult. Clin. Psychol. 71, 419–431. doi: 10.1037/0022-006x.71.3.419 12795567

[ref60] Harnett N. G. Goodman A. M. Knight D. C. (2020). PTSD-related neuroimaging abnormalities in brain function, structure, and biochemistry. Exp. Neurol. 330:113331. doi: 10.1016/j.expneurol.2020.113331 32343956

[ref61] Hartog I. Scherer-Rath M. Kruizinga R. Netjes J. Henriques J. Nieuwkerk P.. (2017). Narrative meaning making and integration: toward a better understanding of the way falling ill influences quality of life. J. Health Psychol. 25, 738–754. doi: 10.1177/1359105317731823 28948830 PMC7221864

[ref62] Hedges L. V. (1981). Distribution theory for Glass’s estimator of effect size and related estimators. J. Educ. Stat. 6:107. doi: 10.2307/1164588

[ref63] Hensel-Dittmann D. Schauer M. Ruf M. Catani C. Odenwald M. Elbert T.. (2011). Treatment of traumatized victims of war and torture: a randomized controlled comparison of narrative exposure therapy and stress inoculation training. Psychother. Psychosom. 80, 345–352. doi: 10.1159/000327253 21829046

[ref64] Hermenau K. Hecker T. Schaal S. Maedl A. Elbert T. (2013). Addressing post-traumatic stress and aggression by means of narrative exposure: a randomized controlled trial with ex-combatants in the eastern DRC. J. Aggression Maltreat. Trauma. 22, 916–934. doi: 10.1080/10926771.2013.824057

[ref65] Higgins J. P. Thompson S. G. Deeks J. J. Altman D. G. (2003). Measuring inconsistency in meta-analyses. BMJ 327, 557–560. doi: 10.1136/bmj.327.7414.557 12958120 PMC192859

[ref66] Hijazi A. M. Lumley M. A. Ziadni M. S. Haddad L. Rapport L. J. Arnetz B. B. (2014). Brief narrative exposure therapy for posttraumatic stress in Iraqi refugees: a preliminary randomized clinical trial. J. Trauma. Stress. 27, 314–322. doi: 10.1002/jts.21922 24866253 PMC4080404

[ref67] Hirsh J. B. Mar R. A. Peterson J. B. (2013). Personal narratives as the highest level of cognitive integration. Behav. Brain Sci. 36, 216–217. doi: 10.1017/s0140525x12002269 23663866

[ref68] Holmes E. A. Crane C. Fennell M. J. Williams J. M. G. (2007). Imagery about suicide in depression—flash-forwards? J. Behav. Ther. Exp. Psychiatry 38, 423–434. doi: 10.1016/j.jbtep.2007.10.004 18037390 PMC2808471

[ref69] Horowitz M. Wilner N. Alvarez W. (1979). Impact of event scale: a measure of subjective stress. Psychosom. Med. 41, 209–218. doi: 10.1097/00006842-197905000-00004 472086

[ref70] Huang M. Schmiedek F. Habermas T. (2020). Only some attempts at meaning making are successful: the role of change-relatedness and positive implications for the self. J. Pers. 89, 175–187. doi: 10.1111/jopy.12573

[ref71] Huang G. Zhang Y. Xiang H. (1992). The Chinese version of the impact of event scale-revised: reliability and validity. Chin. Ment. Health J. Available at: https://pesquisa.bvsalud.org/portal/resource/pt/wpr-585656

[ref72] Ironson G. O’Cleirigh C. Leserman J. Stuetzle R. Fordiani J. Fletcher M.. (2013). Gender-specific effects of an augmented written emotional disclosure intervention on posttraumatic, depressive, and HIV-disease-related outcomes: a randomized, controlled trial. J. Consult. Clin. Psychol. 81, 284–298. doi: 10.1037/a0030814, PMID: 23244367 PMC4465280

[ref73] Jacob N. Neuner F. Maedl A. Schaal S. Elbert T. (2014). Dissemination of psychotherapy for trauma spectrum disorders in postconflict settings: a randomized controlled trial in Rwanda. Psychother. Psychosom. 83, 354–363. doi: 10.1159/000365114 25323203

[ref74] Jaeger J. Lindblom K. M. Parker-Guilbert K. Zoellner L. A. (2014). Trauma narratives: it’s what you say, not how you say it. Psychol. Trauma Theory Res. Pract. Policy 6, 473–481. doi: 10.1037/a0035239 PMC4217123 25379123

[ref75] Janoff-Bulman R. (1992). Shattered assumptions: toward a new psychology of trauma. New York, NY: Free Press.

[ref76] Jelinek L. Randjbar S. Seifert D. Kellner M. Moritz S. (2009). The organization of autobiographical and nonautobiographical memory in posttraumatic stress disorder (PTSD). J. Abnorm. Psychol. 118, 288–298. doi: 10.1037/a0015633 19413404

[ref77] Jelinek L. Stockbauer C. Randjbar S. Kellner M. Ehring T. Moritz S. (2010). Characteristics and organization of the worst moment of trauma memories in posttraumatic stress disorder. Behav. Res. Ther. 48, 680–685. doi: 10.1016/j.brat.2010.03.014 20398896

[ref78] Jobson L. Moradi A. R. Rahimi-Movaghar V. Conway M. A. Dalgleish T. (2014). Culture and the remembering of trauma. Clin. Psychol. Sci. 2, 696–713. doi: 10.1177/2167702614529763

[ref79] Johnson D. M. Ceroni T. L. (2020). “Cognitive behavior therapy for PTSD” in Casebook to the APA clinical practice guideline for the treatment of PTSD. eds. Bufka L. F. Wright C. V. Halfond R. W. (Washington, DC: American Psychological Association), 47–67.

[ref80] Jones C. Harvey A. G. Brewin C. R. (2007). The organisation and content of trauma memories in survivors of road traffic accidents. Behav. Res. Ther. 45, 151–162. doi: 10.1016/j.brat.2006.02.004 16563341

[ref81] Kaplow J. B. Rolon-Arroyo B. Layne C. M. Rooney E. Oosterhoff B. Hill R.. (2020). Validation of the UCLA PTSD reaction index for DSM-5: a developmentally informed assessment tool for youth. J. Am. Acad. Child Adolesc. Psychiatry 59, 186–194. doi: 10.1016/j.jaac.2018.10.019 30953734

[ref82] Kim M. J. Loucks R. A. Palmer A. L. Brown A. C. Solomon K. M. Marchante A. N.. (2011). The structural and functional connectivity of the amygdala: from normal emotion to pathological anxiety. Behav. Brain Res. 223, 403–410. doi: 10.1016/j.bbr.2011.04.025, PMID: 21536077 PMC3119771

[ref83] Kleim B. Ehlers A. Glucksman E. (2007). Early predictors of chronic post-traumatic stress disorder in assault survivors. Psychol. Med. 37, 1457–1467. doi: 10.1017/S0033291707001006 17588274 PMC2829994

[ref84] Kleim B. Horn A. B. Kraehenmann R. Mehl M. R. Ehlers A. (2018). Early linguistic markers of trauma-specific processing predict post-trauma adjustment. Front. Psych. 9:645. doi: 10.3389/fpsyt.2018.00645 PMC6290715 30568607

[ref85] Klein S. B. Robertson T. E. Delton A. W. (2010). Facing the future: memory as an evolved system for planning future acts. Mem. Cogn. 38, 13–22. doi: 10.3758/MC.38.1.13 PMC3553218 19966234

[ref86] Koebach A. Carleial S. Elbert T. Schmitt S. Robjant K. (2021). Treating trauma and aggression with narrative exposure therapy in former child and adult soldiers: a randomized controlled trial in eastern DR Congo. J. Consult. Clin. Psychol. 89, 143–155. doi: 10.1037/ccp0000632 33829803

[ref87] Koenigs M. Grafman J. (2009). Posttraumatic stress disorder: the role of medial prefrontal cortex and amygdala. Neuroscientist 15, 540–548. doi: 10.1177/1073858409333072 19359671 PMC2771687

[ref88] Kolassa I. Illek S. Wilker S. Karabatsiakis A. Elbert T. (2015). “Neurobiological findings in post-traumatic stress disorder” in Evidence based treatments for trauma-related psychological disorders: A practical guide for clinicians. eds. Schnyder U. Cloitre M. (Cham: Springer), 63–86.

[ref89] Krans J. Peeters M. Näring G. Brown A. D. de Bree J. van Minnen A. (2017). Examining temporal alterations in social anxiety disorder and posttraumatic stress disorder: the relation between autobiographical memory, future goals, and current self-views. J. Anxiety Disord. 52, 34–42. doi: 10.1016/j.janxdis.2017.09.007 29031160

[ref90] Kredlow M. A. Fenster R. J. Laurent E. Ressler K. J. Phelps E. A. (2021). Prefrontal cortex, amygdala, and threat processing: implications for PTSD. Neuropsychopharmacology 47, 247–259. doi: 10.1038/s41386-021-01155-7, PMID: 34545196 PMC8617299

[ref91] Lang P. (1977). Imagery in therapy: an information processing analysis of fear. Behav. Ther. 8, 862–886. doi: 10.1016/s0005-7894(77)80157-3 27816081

[ref107] Lechner-Meichsner F. Ehring T. Krüger-Gottschalk A. Morina N. Plankl C. Steil R. (2022). Using imagery rescripting to treat posttraumatic stress disorder in refugees: a case study. Cogn. Behav. Pract. doi: 10.1016/j.cbpra.2022.06.002

[ref92] Lely J. Knipscheer J. W. Moerbeek M. Ter Heide F. J. Van Den Bout J. Kleber R. J. (2019a). Randomised controlled trial comparing narrative exposure therapy with present-centred therapy for older patients with post-traumatic stress disorder. Br. J. Psychiatry 214, 369–377. doi: 10.1192/bjp.2019.59 30957736

[ref93] Lely J. Smid G. E. Jongedijk R. A. Knipscheer J. W. Kleber R. J. (2019b). The effectiveness of narrative exposure therapy: a review, meta-analysis and meta-regression analysis. Eur. J. Psychotraumatol. 10:1550344. doi: 10.1080/20008198.2018.1550344, PMID: 31007868 PMC6450467

[ref94] Lindblom K. M. Gray M. J. (2010). Relationship closeness and trauma narrative detail: a critical analysis of betrayal trauma theory. Appl. Cogn. Psychol. 24, 1–19. doi: 10.1002/acp.1547

[ref95] Lin-Stephens S. (2020). Visual stimuli in narrative-based interventions for adult anxiety: a systematic review. Anxiety Stress Coping 33, 281–298. doi: 10.1080/10615806.2020.1734575 32126824

[ref96] Loganovsky K. N. Zdanevich N. A. (2013). Cerebral basis of posttraumatic stress disorder following the Chernobyl disaster. CNS Spectr. 18, 95–102. doi: 10.1017/S109285291200096X 23445934

[ref97] Lyoo I. K. Kim J. Yoon S. Hwang J. Bae S. Kim D. (2011). The neurobiological role of the dorsolateral prefrontal cortex in recovery from trauma. Arch. Gen. Psychiatry 68:701. doi: 10.1001/archgenpsychiatry.2011.70 21727254

[ref98] Marin K. A. Shkreli A. (2019). An examination of trauma narratives: narrative rumination, self-reflection, and identity in young adulthood. J. Adolesc. 76, 139–151. doi: 10.1016/j.adolescence.2019.08.007 31479894

[ref99] Marks E. H. Franklin A. R. Zoellner L. A. (2018). Can't get it out of my mind: a systematic review of predictors of intrusive memories of distressing events. Psychol. Bull. 144, 584–640. doi: 10.1037/bul0000132 29553763 PMC5938103

[ref100] May C. L. Wisco B. E. (2016). Defining trauma: how level of exposure and proximity affect risk for posttraumatic stress disorder. Psychol. Trauma Theory Res. Pract. Policy 8, 233–240. doi: 10.1037/tra0000077 26390110

[ref101] McAdams D. P. (2019). “First we invented stories, then they changed us”: the evolution of narrative identity. ESIC 3, 1–18. doi: 10.26613/esic.3.1.110

[ref102] McGaugh J. L. (2004). The amygdala modulates the consolidation of memories of emotionally arousing experiences. Annu. Rev. Neurosci. 27, 1–28. doi: 10.1146/annurev.neuro.27.070203.144157, PMID: 15217324

[ref103] McIntire L. (2014). Effects of narrative writing and post-writing processing instructions on PTSD. The University of Mississippi ProQuest, Dissertations Publishing. 3639440. Available at: https://egrove.olemiss.edu/etd/1140/

[ref104] McLean C. P. Levy H. C. Miller M. L. Tolin D. F. (2022). Exposure therapy for PTSD: a meta-analysis. Clin. Psychol. Rev. 91:102115. doi: 10.1016/j.cpr.2021.102115 34954460

[ref105] McNally R. J. (2003). Progress and controversy in the study of posttraumatic stress disorder. Annu. Rev. Psychol. 54, 229–252. doi: 10.1146/annurev.psych.54.101601.145112 12172002

[ref106] McNally R. J. (2022). Are memories of sexual trauma fragmented? A post publication discussion among Richard J. McNally, Dorthe Berntsen, Chris R. Brewin and David C. Rubin. Memory 30, 658–660. doi: 10.1080/09658211.2022.2061135 35392773

[ref108] Merckelbach H. Langeland W. De Vries G. Draijer N. (2014). Symptom overreporting obscures the dose–response relationship between trauma severity and symptoms. Psychiatry Res. 217, 215–219. doi: 10.1016/j.psychres.2014.03.018, PMID: 24704260

[ref109] Mollica R. F. Caspi-Yavin Y. Bollini P. Truong T. Tor S. Lavelle J. (1992). The Harvard trauma questionnaire: validating a cross-cultural instrument for measuring torture, trauma, and posttraumatic stress disorder in Indochinese refugees. J. Nerv. Ment. Dis. 180, 111–116. doi: 10.1097/00005053-199202000-00008 1737972

[ref110] Morath J. Gola H. Sommershof A. Hamuni G. Kolassa S. Catani C.. (2014a). The effect of trauma-focused therapy on the altered T cell distribution in individuals with PTSD: evidence from a randomized controlled trial. J. Psychiatr. Res. 54, 1–10. doi: 10.1016/j.jpsychires.2014.03.016 24726027

[ref111] Morath J. Moreno-Villanueva M. Hamuni G. Kolassa S. Ruf-Leuschner M. Schauer M.. (2014b). Effects of psychotherapy on DNA Strand break accumulation originating from traumatic stress. Psychother. Psychosom. 83, 289–297. doi: 10.1159/000362739, PMID: 25116690

[ref112] Moreira A. Moreira A. C. Rocha J. C. (2020). Randomized controlled trial: cognitive-narrative therapy for IPV victims. J. Interpers. Violence 37, NP2998–NP3014. doi: 10.1177/0886260520943719 32755265

[ref113] Muijnck D. (2022). Narrative, memory and PTSD. A case study of autobiographical narration after trauma. Eur. J. Life Writ. 11, AN75–AN95. doi: 10.21827/ejlw.11.38659

[ref9004] Murray J. Ehlers A. Mayou R. A. (2002). Dissociation and post-traumatic stress disorder: two prospective studies of road traffic accident survivors. Br. J. Psych: the journal of mental science, 180, 363–368. doi: 10.1192/bjp.180.4.363 11925361

[ref114] Neuner F. Kurreck S. Ruf M. Odenwald M. Elbert T. Schauer M. (2009). Can asylum-seekers with posttraumatic stress disorder be successfully treated? A randomized controlled pilot study. Cogn. Behav. Ther. 39, 81–91. doi: 10.1080/16506070903121042 19816834

[ref115] Neuner F. Onyut P. L. Ertl V. Odenwald M. Schauer E. Elbert T. (2008). Treatment of posttraumatic stress disorder by trained lay counselors in an African refugee settlement: a randomized controlled trial. J. Consult. Clin. Psychol. 76, 686–694. doi: 10.1037/0022-006x.76.4.686 18665696

[ref116] Neuner F. Schauer M. Klaschik C. Karunakara U. Elbert T. (2004). A comparison of narrative exposure therapy, supportive counseling, and psychoeducation for treating posttraumatic stress disorder in an African refugee settlement. J. Consult. Clin. Psychol. 72, 579–587. doi: 10.1037/0022-006x.72.4.579 15301642

[ref117] Ng R. M. Di Simplicio M. McManus F. Kennerley H. Holmes E. A. (2016). ‘Flash-forwards’ and suicidal ideation: a prospective investigation of mental imagery, entrapment and defeat in a cohort from the Hong Kong Mental Morbidity Survey. Psychiatry Res. 246, 453–460. doi: 10.1016/j.psychres.2016.10.018 27792974

[ref118] Olkin I. Dahabreh I. J. Trikalinos T. A. (2012). GOSH – a graphical display of study heterogeneity. Res. Synth. Methods 3, 214–223. doi: 10.1002/jrsm.1053 26062164

[ref119] Orang T. Ayoughi S. Moran J. K. Ghaffari H. Mostafavi S. Rasoulian M.. (2018). The efficacy of narrative exposure therapy in a sample of Iranian women exposed to ongoing intimate partner violence-a randomized controlled trial. Clin. Psychol. Psychother. 25, 827–841. doi: 10.1002/cpp.2318 30079583

[ref120] Pace T. W. Heim C. M. (2011). A short review on the psychoneuroimmunology of posttraumatic stress disorder: from risk factors to medical comorbidities. Brain Behav. Immun. 25, 6–13. doi: 10.1016/j.bbi.2010.10.003 20934505

[ref121] Paré D. Duvarci S. (2012). Amygdala microcircuits mediating fear expression and extinction. Curr. Opin. Neurobiol. 22, 717–723. doi: 10.1016/j.conb.2012.02.014 22424846 PMC3380167

[ref122] Park C. L. (2010). Making sense of the meaning literature: an integrative review of meaning making and its effects on adjustment to stressful life events. Psychol. Bull. 136, 257–301. doi: 10.1037/a0018301 20192563

[ref123] Park C. L. (2022). Meaning making following trauma. Front. Psychol. 13:844891. doi: 10.3389/fpsyg.2022.844891 35401307 PMC8984472

[ref124] Park C. L. Boals A. (2021). “Current assessment and interpretation of perceived post-traumatic growth” in Redesigning research on post-traumatic growth. eds. Infurna F. J. Jayawickreme E. (Oxford: Oxford University Press), 12–27.

[ref125] Park J. K. Park J. Elbert T. Kim S. J. (2020). Effects of narrative exposure therapy on posttraumatic stress disorder, depression, and insomnia in traumatized North Korean refugee youth. J. Trauma. Stress. 33, 353–359. doi: 10.1002/jts.22492 32216143 PMC7317474

[ref126] Phelps E. A. LeDoux J. E. (2005). Contributions of the amygdala to emotion processing: from animal models to human behavior. Neuron 48, 175–187. doi: 10.1016/j.neuron.2005.09.025 16242399

[ref127] Qian J. Sun S. Zhou X. Wu M. Yu X. (2021). Effects of an expressive writing intervention in Chinese women undergoing pregnancy termination for fetal abnormality: a randomized controlled trial. Midwifery 103:103104. doi: 10.1016/j.midw.2021.103104 34348194

[ref128] Quintana M. I. Mari J. d. J. Ribeiro W. S. Jorge M. R. Andreoli S. B. (2012). Accuracy of the composite international diagnostic interview (CIDI 2.1) for diagnosis of post-traumatic stress disorder according to DSM-IV criteria. Cad. Saude Publica. 28, 1312–1318. doi: 10.1590/s0102-311x2012000700009 22729261

[ref129] Raeder R. (2022). Narrative, memory, and mental health: A critical review of autobiographical memory architecture in PTSD (Unpublished master’s dissertation). The Institute of Psychiatry, Psychology & Neuroscience, King’s College London.

[ref130] Ramasubramanian M. Patel D. Turner M. R. Ybarra V. (2022). The influence of life narrative themes on resilience and life outcomes. Personal. Individ. Differ. 185:111235. doi: 10.1016/j.paid.2021.111235

[ref131] Rauch S. L. Shin L. M. Wright C. I. (2003). Neuroimaging studies of amygdala function in anxiety disorders. Ann. N. Y. Acad. Sci. 985, 389–410. doi: 10.1111/j.1749-6632.2003.tb07096.x 12724173

[ref132] Resick P. A. (1992). “Cognitive treatment of a crime-related PTSD” in Aggression and violence throughout the lifespan. eds. Peters R. D. McMahon R. J. Quinsey V. L. (Newbury Park, CA: Sage), 171–191.

[ref133] Resick P. A. Monson C. M. Chard K. M. (2017). Cognitive processing therapy for PTSD: a comprehensive manual. New York City: Guilford Press.

[ref134] Resick P. A. Schnicke M. K. (1992). Cognitive processing therapy for sexual assault victims. J. Consult. Clin. Psychol. 60, 748–756. doi: 10.1037//0022-006x.60.5.748 1401390

[ref135] Resick P.A. Schnicke M.K. (1993). Cognitive processing therapy for rape victims: a treatment manual. Newbury Park, CA: Sage.

[ref136] Rizvi S. L. Vogt D. S. Resick P. A. (2009). Cognitive and affective predictors of treatment outcome in cognitive processing therapy and prolonged exposure for posttraumatic stress disorder. Behav. Res. Ther. 47, 737–743. doi: 10.1016/j.brat.2009.06.003 19595295 PMC3467002

[ref137] Robjant K. Koebach A. Schmitt S. Chibashimba A. Carleial S. Elbert T. (2019). The treatment of posttraumatic stress symptoms and aggression in female former child soldiers using adapted narrative exposure therapy – a RCT in eastern Democratic Republic of Congo. Behav. Res. Ther. 123:103482. doi: 10.1016/j.brat.2019.103482 31639529

[ref138] Roediger H. L. (1990). Implicit memory: retention without remembering. Am. Psychol. 45:1043. doi: 10.1037//0003-066x.45.9.1043 2221571

[ref139] Rubin D. C. (Ed.). (1988). Autobiographical memory. Cambridge: Cambridge University Press.

[ref140] Schacter D. L. (2019). Implicit memory, constructive memory, and imagining the future: a career perspective. Perspect. Psychol. Sci. 14, 256–272. doi: 10.1177/1745691618803640 30517833 PMC6402953

[ref141] Schacter D. L. Addis D. R. (2007). The cognitive neuroscience of constructive memory: remembering the past and imagining the future. Philos. Trans. R. Soc. Lond. Ser. B Biol. Sci. 362, 773–786. doi: 10.1098/rstb.2007.2087, PMID: 17395575 PMC2429996

[ref142] Schacter D. L. Tulving E. (1994). Memory systems 1994 1st ed. New York: Bradford Book.

[ref143] Schaefer S. M. Morozink Boylan J. van Reekum C. M. Lapate R. C. Norris C. J. Ryff C. D.. (2013). Purpose in life predicts better emotional recovery from negative stimuli. PLoS One 8:e80329. doi: 10.1371/journal.pone.0080329, PMID: 24236176 PMC3827458

[ref145] Schauer M. Neuner F. Elbert T. (2011). Narrative exposure therapy: a short-term treatment for traumatic stress disorders 2nd. Boston, MA: Hogrefe Publishing.

[ref146] Schneider A. Pfeiffer A. Conrad D. Elbert T. Kolassa I. T. Wilker S. (2020). Does cumulative exposure to traumatic stressors predict treatment outcome of community-implemented exposure-based therapy for PTSD? Eur. J. Psychotraumatol. 11:1789323. doi: 10.1080/20008198.2020.1789323 33062203 PMC7534285

[ref147] Schönfeld S. Ehlers A. (2017). Posttraumatic stress disorder and autobiographical memories in everyday life. Clin. Psychol. Sci. 5, 325–340. doi: 10.1177/2167702616688878 28781928 PMC5528199

[ref148] Schultebraucks K. Duesenberg M. Di Simplicio M. Holmes E. A. Roepke S. (2020). Suicidal imagery in borderline personality disorder and major depressive disorder. J. Personal. Disord. 34, 546–564. doi: 10.1521/pedi_2019_33_406 30785849

[ref149] Shapiro F. Forrest M. (2001). EMDR: eye movement desensitisation and reprocessing. New York City: Guilford.

[ref150] Sheldon S. Fenerci C. Gurguryan L. (2019). A neurocognitive perspective on the forms and functions of autobiographical memory retrieval. Front. Syst. Neurosci. 13:4. doi: 10.3389/fnsys.2019.00004, PMID: 30760984 PMC6361758

[ref151] Shin L. M. Rauch S. L. Pitman R. K. (2006). Amygdala, medial prefrontal cortex, and hippocampal function in PTSD. Ann. N. Y. Acad. Sci. 1071, 67–79. doi: 10.1196/annals.1364.007 16891563

[ref152] Sigurdsson T. Doyère V. Cain C. K. LeDoux J. E. (2007). Long-term potentiation in the amygdala: a cellular mechanism of fear learning and memory. Neuropharmacology 52, 215–227. doi: 10.1016/j.neuropharm.2006.06.022 16919687

[ref153] Simons J. S. Spiers H. J. (2003). Prefrontal and medial temporal lobe interactions in long-term memory. Nat. Rev. Neurosci. 4, 637–648. doi: 10.1038/nrn1178 12894239

[ref154] Singer J. A. Blagov P. S. (2004). “Self-defining memories, narrative identity, and psychotherapy: a conceptual model, empirical investigation, and case report” in The handbook of narrative and psychotherapy: practice, theory, and research. eds. Angus L. E. McLeod J. (Thousand Oaks, CA: Sage Publications, Inc.), 229–246.

[ref155] Singer J. A. Blagov P. Berry M. Oost K. M. (2013). Self-defining memories, scripts, and the life story: narrative identity in personality and psychotherapy. J. Pers. 81, 569–582. doi: 10.1111/jopy.12005 22925032

[ref156] Sipahi L. Uddin M. Hou Z. G. Aiello A. E. Koenen K. C. Galea S.. (2014). Ancient evolutionary origins of epigenetic regulation associated with posttraumatic stress disorder. Front. Hum. Neurosci. 8:284. doi: 10.3389/fnhum.2014.00284 24860472 PMC4026723

[ref157] Sloan D. M. Marx B. P. Bovin M. J. Feinstein B. A. Gallagher M. W. (2012). Written exposure as an intervention for PTSD: a randomized clinical trial with motor vehicle accident survivors. Behav. Res. Ther. 50, 627–635. doi: 10.1016/j.brat.2012.07.001 22863540 PMC3433579

[ref158] Sloan D. M. Marx B. P. Greenberg E. M. (2011). A test of written emotional disclosure as an intervention for posttraumatic stress disorder. Behav. Res. Ther. 49, 299–304. doi: 10.1016/j.brat.2011.02.001, PMID: 21367400 PMC3898617

[ref159] Sloan D. M. Marx B. P. Lee D. J. Resick P. A. (2018). A brief exposure-based treatment vs cognitive processing therapy for posttraumatic stress disorder: a randomized noninferiority clinical trial. JAMA Psychiat. 75:233. doi: 10.1001/jamapsychiatry.2017.4249 PMC5843538 29344631

[ref160] Sloan D. M. Marx B. P. Resick P. A. Young-McCaughan S. Dondanville K. A. Straud C. L.. (2022). Effect of written exposure therapy vs cognitive processing therapy on increasing treatment efficiency among military service members with posttraumatic stress disorder. JAMA Netw. Open 5:e2140911. doi: 10.1001/jamanetworkopen.2021.40911 35015065 PMC8753496

[ref161] Squire L. R. (1992). Declarative and nondeclarative memory: multiple brain systems supporting learning and memory. J. Cogn. Neurosci. 4, 232–243. doi: 10.1162/jocn.1992.4.3.232 23964880

[ref162] Steil R. Fischer A. C. Gutermann J. Rosner R. (2022). Mental imagery in adolescent PTSD patients after child abuse: a comparison with matched healthy controls. BMC Psychiatry 22:64. doi: 10.1186/s12888-022-03706-8 35086493 PMC8793273

[ref164] Stevens J. S. Reddy R. Kim Y. Van Rooij S. J. Ely T. D. Hamann S.. (2018). Episodic memory after trauma exposure: medial temporal lobe function is positively related to re-experiencing and inversely related to negative affect symptoms. NeuroImage Clin. 17, 650–658. doi: 10.1016/j.nicl.2017.11.016 29204343 PMC5709292

[ref165] Suddendorf T. Corballis M. C. (1997). Mental time travel and the evolution of the human mind. Genet. Soc. Gen. Psychol. Monogr. 123, 133–167. 9204544

[ref166] Sutherland K. Bryant R. A. (2005). Self-defining memories in post-traumatic stress disorder. Br. J. Clin. Psychol. 44, 591–598. doi: 10.1348/014466505X64081 16368036

[ref167] Sutin A. R. Luchetti M. Aschwanden D. Stephan Y. Terracciano A. (2021). Sense of purpose in life, cognitive function, and the phenomenology of autobiographical memory. Memory 29, 1126–1135. doi: 10.1080/09658211.2021.1966472 34460357 PMC8883603

[ref168] Talarico J. M. Rubin D. C. (2003). Confidence, not consistency, characterizes flashbulb memories. Psychol. Sci. 14, 455–461. doi: 10.1111/1467-9280.02453 12930476

[ref169] Thielemann J. Kasparik B. König J. Unterhitzenberger J. Rosner R. (2022). A systematic review and meta-analysis of trauma-focused cognitive behavioral therapy for children and adolescents. Child Abuse Negl. 134:105899. doi: 10.1016/j.chiabu.2022.105899 36155943

[ref170] Thompson-Hollands J. Marx B. P. Sloan D. M. (2019). Brief novel therapies for PTSD: written exposure therapy. Curr. Treat. Options Oncol. 6, 99–106. doi: 10.1007/s40501-019-00168-w PMC6594556 31245252

[ref171] Tulving E. (1972). “Episodic and semantic memory” in Organization of memory. eds. Tulving E. Donaldson W. (London: Academic Press)

[ref172] Tulving E. Craik F. I. M. (2000). The Oxford handbook of memory. Oxford: Oxford University Press.

[ref173] Van Le Q. Isbell L. A. Matsumoto J. Le V. B. Nishimaru H. Hori E.. (2016). Snakes elicit earlier, and monkey faces, later, gamma oscillations in macaque pulvinar neurons. Sci. Rep. 6:20595. doi: 10.1038/srep20595 26854087 PMC4744932

[ref174] Van Le Q. Isbell L. A. Matsumoto J. Nguyen M. Hori E. Maior R. S.. (2013). Pulvinar neurons reveal neurobiological evidence of past selection for rapid detection of snakes. Proc. Natl. Acad. Sci. U. S. A. 110, 19000–19005. doi: 10.1073/pnas.1312648110 24167268 PMC3839741

[ref175] Vevea J. L. Hedges L. V. (1995). A general linear model for estimating effect size in the presence of publication bias. Psychometrika 60, 419–435. doi: 10.1007/BF02294384

[ref176] Viechtbauer W. (2010). Conducting meta-analyses in R with the metafor package. J. Stat. Softw. 36, 1–48. doi: 10.18637/jss.v036.i03

[ref177] Weathers F. W. Bovin M. J. Lee D. J. Sloan D. M. Schnurr P. P. Kaloupek D. G.. (2018). The clinician-administered PTSD scale for DSM-5 (CAPS-5): development and initial psychometric evaluation in military veterans. Psychol. Assess. 30, 383–395. doi: 10.1037/pas0000486 28493729 PMC5805662

[ref178] Wei Y. Chen S. (2021). Narrative exposure therapy for posttraumatic stress disorder: a meta-analysis of randomized controlled trials. Psychol. Trauma Theory Res. Pract. Policy 13, 877–884. doi: 10.1037/tra0000922 33617284

[ref179] Wickham H. (2016). ggplot2: Elegant graphics for data analysis. Springer-Verlag New York.

[ref180] Wilker S. Vukojevic V. Schneider A. Pfeiffer A. Inerle S. Pauly M.. (2023). Epigenetics of traumatic stress: the association of NR3C1 methylation and posttraumatic stress disorder symptom changes in response to narrative exposure therapy. Transl. Psychiatry 13:14. doi: 10.1038/s41398-023-02316-6 36658116 PMC9852425

[ref181] Wilkins C. Clayton N. (2019). Reflections on the spoon test. Neuropsychologia 134:107221. doi: 10.1016/j.neuropsychologia.2019.107221, PMID: 31586552

[ref182] Williams H. L. Conway M. A. Cohen G. (2008). “Autobiographical memory” in Memory in the real world. eds. Cohen G. Conway M. A. (London: Psychology Press), 21–90.

[ref9005] Willingham D. B. (1997). Systems of memory in the human brain. Neuron 18, 5–8. doi: 10.1016/s0896-6273(01)80040-4 9010199

[ref183] Womersley J. Xulu K. Sommer J. Hinsberger M. Elbert T. Weierstall R.. (2020). P.698 DNA methylation correlates of narrative exposure therapy for forensic offender rehabilitation in trauma-exposed men with appetitive aggression. Eur. Neuropsychopharmacol. 40, S397–S398. doi: 10.1016/j.euroneuro.2020.09.516

[ref184] Zakarian R. J. McDevitt-Murphy M. E. Bellet B. W. Neimeyer R. A. Burke L. A. (2019). Relations among meaning making, PTSD, and complicated grief following homicide loss. J. Loss Trauma 24, 279–291. doi: 10.1080/15325024.2019.1565111

[ref185] Zang Y. Hunt N. Cox T. (2013). A randomised controlled pilot study: the effectiveness of narrative exposure therapy with adult survivors of the Sichuan earthquake. BMC Psychiatry 13:41. doi: 10.1186/1471-244x-13-41 23363689 PMC3570314

[ref186] Zang Y. Hunt N. Cox T. (2014). Adapting narrative exposure therapy for Chinese earthquake survivors: a pilot randomised controlled feasibility study. BMC Psychiatry 14:262. doi: 10.1186/s12888-014-0262-3, PMID: 25927297 PMC4189751

[ref187] Zolfa R. Moradi A. Mahdavi M. Parhoon H. Parhoon K. Jobson L. (2022). Feasibility and acceptability of written exposure therapy in addressing posttraumatic stress disorder in Iranian patients with breast cancer. Psycho-Oncology 32, 68–76. doi: 10.1002/pon.6037 36116086

