# Supplementary material for: Narrative-based autobiographical memory interventions for PTSD: a meta-analysis of randomized controlled trials
Source: Front Psychol. 2023 Sep 27;14:1215225. doi: 10.3389/fpsyg.2023.1215225 (PMC10565228; doi:10.3389/fpsyg.2023.1215225)
Supplement: Supplementary file 1 [file Data_Sheet_1.docx]

Supplementary Material

Narrative-Based Autobiographical Memory Interventions for PTSD: A Meta-Analysis of Randomized Controlled Trials

Robert Raeder^1^, Nicola S Clayton^1,†^, Markus Boeckle,^2,3,†,*^

^1^Department of Psychology, University of Cambridge, Cambridge, UK

^2^Karl Landsteiner University of Health Sciences, Krems, Austria

^3^Department of Transitory Psychiatry, University Hospital Tulln, Tulln, Austria

†Joint senior authorship

*** Correspondence:** Markus Boeckle, [markus.boeckle@gmail.com](mailto:markus.boeckle@gmail.com)

# Risk of Bias Assessment

**Table 1.** The following table outlines the risk of bias for all 38 studies that met the full inclusion criteria for the present meta-analysis according to the revised Joanna Briggs Institute (JBI) critical appraisal tool for the assessment of risk of bias for randomized controlled trials (RCTs). The JBI tool focuses on assessing the methodological quality of individual studies within systematic reviews and meta-analyses by evaluating study design, methodology, data collection, and data analysis. It is helpful when evaluating the internal validity of studies and determining how much confidence can be placed in their findings. While the commonly used GRADE framework considers various factors in addition to risk of bias (such as inconsistency, imprecision, and indirectness), the JBI tool may be more comprehensive in evaluating risk of bias for each individual study. Therefore, due to the heterogeneity across various types of interventions in included studies, this meta-analysis prioritized a detailed examination of the potential sources of bias in each individual study by using the JBI checklist to ensure the robustness of findings.

In the above table, the columns are reflective of the following questions:

# Was true randomization used for assignment of participants to treatment groups?

# Was allocation to treatment groups concealed?

# Were treatment groups similar at the baseline?

# Were participants blind to treatment assignment?

# Were those delivering treatment blind to treatment assignment?

# Were outcomes assessors blind to treatment assignment?

# Were treatment groups treated identically other than the intervention of interest?

# Was follow up complete and if not, were differences between groups in terms of their follow up adequately described and analyzed?

# Were participants analyzed in the groups to which they were randomized?

# Were outcomes measured in the same way for treatment groups?

# Were outcomes measured in a reliable way?

# Was appropriate statistical analysis used?

# Was the trial design appropriate, and any deviations from the standard RCT design (individual randomization, parallel groups) accounted for in the conduct and analysis of the trial?

References

Adenauer, H., Catani, C., Gola, H., Keil, J., Ruf, M., Schauer, M., et al. (2011). Narrative exposure therapy for PTSD increases top-down processing of aversive stimuli – evidence from a randomized controlled treatment trial. *BMC Neurosci.* 12:127. doi: 10.1186/1471-2202-12-127

Alessandri, F. T. (2017). Testing a brief directive intervention to reduce symptoms associated with trauma. *eGrove* Available at: https://egrove.olemiss.edu/etd/829/

Alghamdi, M., Hunt, N., and Thomas, S. (2015). The effectiveness of narrative exposure therapy with traumatised firefighters in Saudi Arabia: a randomized controlled study. *Behav. Res. Ther.* 66, 64–71. doi: 10.1016/j.brat.2015.01.008

Al-Hadethe, A., Hunt, N., Al-Qaysi, G., and Thomas, S. (2015). Randomised controlled study comparing two psychological therapies for posttraumatic stress disorder (PTSD): emotional freedom techniques (EFT) vs. Narrative Exposure Therapy (NET). *J. Trauma. Stress Disord. Treat.* 4:4. doi: 10.4172/2324-8947.1000145

Bichescu, D., Neuner, F., Schauer, M., and Elbert, T. (2007). Narrative exposure therapy for political imprisonment-related chronic posttraumatic stress disorder and depression. *Behav. Res. Ther.* 45, 2212–2220. doi: 10.1016/j.brat.2006.12.006

Brady, F., Chisholm, A., Walsh, E., Ottisova, L., Bevilacqua, L., Mason, C., et al. (2021). Narrative exposure therapy for survivors of human trafficking: feasibility randomised controlled trial. *BJPsych. Open* 7:e196. doi: 10.1192/bjo.2021.1029

Fan, Y., Shi, Y., Zhang, J., Sun, D., Wang, X., Fu, G., et al. (2021). The effects of narrative exposure therapy on COVID-19 patients with post-traumatic stress symptoms: a randomized controlled trial. *J. Affect. Disord.* 293, 141–147. doi: 10.1016/j.jad.2021.06.019

Gensichen, J., Friemel, C., Schmidt, K., Sanftenberg, L., Dohmann, J., Reips, U. D., et al. (2022). A primary care-based narrative exposure therapy on patients with post- traumatic stress disorder following intensive care. *Am. J. Respir. Crit. Care Med.* 205:A5363. doi: 10.1164/ajrccm-conference.2022.205.1_meetingabstracts.a5363

Gofman, M., Kivity, Y., Bar-Kalifa, E., Vidan, Z., Ohayon, I. H., Tuval-Mashiach, R., et al. (2021). Narrative reconstruction as an intervention for posttraumatic stress disorder: a pilot delayed intervention quasi-randomized controlled trial. *J. Trauma. Stress.* 34, 92–103. doi: 10.1002/jts.22537

Gray, R., Budden-Potts, D., and Bourke, F. (2017). Reconsolidation of traumatic memories for PTSD: a randomized controlled trial of 74 male veterans. *Psychother. Res.* 29, 621–639. doi: 10.1080/10503307.2017.1408973

Hensel-Dittmann, D., Schauer, M., Ruf, M., Catani, C., Odenwald, M., Elbert, T., et al. (2011). Treatment of traumatized victims of war and torture: a randomized controlled comparison of narrative exposure therapy and stress inoculation training. *Psychother. Psychosom.* 80, 345–352. doi: 10.1159/000327253

Hermenau, K., Hecker, T., Schaal, S., Maedl, A., and Elbert, T. (2013). Addressing post-traumatic stress and aggression by means of narrative exposure: a randomized
controlled trial with ex-combatants in the eastern DRC. *J. Aggression Maltreat. Trauma.* 22, 916–934. doi: 10.1080/10926771.2013.824057

Hijazi, A. M., Lumley, M. A., Ziadni, M. S., Haddad, L., Rapport, L. J., and Arnetz, B. B. (2014). Brief narrative exposure therapy for posttraumatic stress in Iraqi refugees: a preliminary randomized clinical trial. *J. Trauma. Stress.* 27, 314–322. doi: 10.1002/jts.21922

Ironson, G., O’Cleirigh, C., Leserman, J., Stuetzle, R., Fordiani, J., Fletcher, M., et al. (2013). Gender-specific effects of an augmented written emotional disclosure intervention on posttraumatic, depressive, and HIV-disease-related outcomes: a randomized, controlled trial. *J. Consult. Clin. Psychol.* 81, 284–298. doi: 10.1037/ a0030814

Jacob, N., Neuner, F., Maedl, A., Schaal, S., and Elbert, T. (2014). Dissemination of psychotherapy for trauma spectrum disorders in postconflict settings: a randomized controlled trial in Rwanda. *Psychother. Psychosom.* 83, 354–363. doi: 10.1159/000365114

Koebach, A., Carleial, S., Elbert, T., Schmitt, S., and Robjant, K. (2021). Treating trauma and aggression with narrative exposure therapy in former child and adult soldiers: a randomized controlled trial in eastern DR Congo. *J. Consult. Clin. Psychol.* 89, 143–155. doi: 10.1037/ccp0000632

Lely, J., Knipscheer, J. W., Moerbeek, M., Ter Heide, F. J., Van Den Bout, J., and Kleber, R. J. (2019). Randomised controlled trial comparing narrative exposure therapy with present-centred therapy for older patients with post-traumatic stress disorder. *Br. J. Psychiatry* 214, 369–377. doi: 10.1192/bjp.2019.59

McIntire, L. (2014). Effects of narrative writing and post-writing processing instructions on PTSD. The University of Mississippi ProQuest, Dissertations Publishing. 3639440. Available at: https://egrove.olemiss.edu/etd/1140/

Morath, J., Gola, H., Sommershof, A., Hamuni, G., Kolassa, S., Catani, C., et al. (2014a). The effect of trauma-focused therapy on the altered T cell distribution in individuals with PTSD: evidence from a randomized controlled trial. *J. Psychiatr. Res.* 54, 1–10. doi: 10.1016/j.jpsychires.2014.03.016

Morath, J., Moreno-Villanueva, M., Hamuni, G., Kolassa, S., Ruf-Leuschner, M., Schauer, M., et al. (2014b). Effects of psychotherapy on DNA Strand break accumulation originating from traumatic stress. *Psychother. Psychosom.* 83, 289–297. doi: 10.1159/000362739

Moreira, A., Moreira, A. C., and Rocha, J. C. (2020). Randomized controlled trial: cognitive-narrative therapy for IPV victims. *J. Interpers. Violence* 37, NP2998–NP3014. doi: 10.1177/0886260520943719

Neuner, F., Kurreck, S., Ruf, M., Odenwald, M., Elbert, T., and Schauer, M. (2009). Can asylum-seekers with posttraumatic stress disorder be successfully treated? A 2737 randomized controlled pilot study. *Cogn. Behav. Ther.* 39, 81–91. doi:10.1080/16506070903121042

Neuner, F., Onyut, P. L., Ertl, V., Odenwald, M., Schauer, E., and Elbert, T. (2008). Treatment of posttraumatic stress disorder by trained lay counselors in an African refugee settlement: a randomized controlled trial. *J. Consult. Clin. Psychol.* 76, 686–694. doi: 10.1037/0022-006x.76.4.686

Neuner, F., Schauer, M., Klaschik, C., Karunakara, U., and Elbert, T. (2004). A comparison of narrative exposure therapy, supportive counseling, and psychoeducation for treating posttraumatic stress disorder in an African refugee settlement. *J. Consult. Clin. Psychol.* 72, 579–587. doi: 10.1037/0022-006x.72.4.579

Orang, T., Ayoughi, S., Moran, J. K., Ghaffari, H., Mostafavi, S., Rasoulian, M., et al. (2018). The efficacy of narrative exposure therapy in a sample of Iranian women exposed to ongoing intimate partner violence-a randomized controlled trial. *Clin. Psychol. Psychother.* 25, 827–841. doi: 10.1002/cpp.2318

Park, J. K., Park, J., Elbert, T., and Kim, S. J. (2020). Effects of narrative exposure therapy on posttraumatic stress disorder, depression, and insomnia in traumatized North Korean refugee youth. *J. Trauma. Stress.* 33, 353–359. doi: 10.1002/jts.22492

Qian, J., Sun, S., Zhou, X., Wu, M., and Yu, X. (2021). Effects of an expressive writing intervention in Chinese women undergoing pregnancy termination for fetal abnormality: a randomized controlled trial. *Midwifery* 103:103104. doi: 10.1016/j.midw.2021.103104

Robjant, K., Koebach, A., Schmitt, S., Chibashimba, A., Carleial, S., and Elbert, T. (2019). The treatment of posttraumatic stress symptoms and aggression in female former child soldiers using adapted narrative exposure therapy – a RCT in eastern Democratic Republic of Congo. *Behav. Res. Ther.* 123:103482. doi: 10.1016/j.brat.2019.103482

Schauer, M., Elbert, T., Gotthardt, S., Rockstroh, B., Odenwald, M., and Neuner, F. (2006). Wiedererfahrung durch Psychotherapie modifiziert Geist und Gehirn*. *Verhaltenstherapie* 16, 96–103. doi: 10.1159/000093195

Sloan, D. M., Marx, B. P., Bovin, M. J., Feinstein, B. A., and Gallagher, M. W. (2012). Written exposure as an intervention for PTSD: a randomized clinical trial with motor vehicle accident survivors. *Behav. Res. Ther.* 50, 627–635. doi: 10.1016/j.brat.2012.07.001

Sloan, D. M., Marx, B. P., and Greenberg, E. M. (2011). A test of written emotional disclosure as an intervention for posttraumatic stress disorder. *Behav. Res. Ther.* 49, 299–304. doi: 10.1016/j.brat.2011.02.001

Sloan, D. M., Marx, B. P., Lee, D. J., and Resick, P. A. (2018). A brief exposure-based treatment vs cognitive processing therapy for posttraumatic stress disorder: a randomized 2840 noninferiority clinical trial. *JAMA Psychiat.* 75:233. doi: 10.1001/jamapsychiatry.2017.4249

Sloan, D. M., Marx, B. P., Resick, P. A., Young-McCaughan, S., Dondanville, K. A., Straud, C. L., et al. (2022). Effect of written exposure therapy vs cognitive processing therapy on increasing treatment efficiency among military service members with posttraumatic stress disorder. *JAMA Netw. Open* 5:e2140911. doi: 10.1001/jamanetworkopen.2021.40911

Stenmark, H., Catani, C., Neuner, F., Elbert, T., and Holen, A. (2013). Treating PTSD in refugees and asylum seekers within the general health care system. A randomized controlled multicenter study. *Behav. Res. Ther.* 51, 641–647. doi: 10.1016/j.brat.2013.07.002

Womersley, J., Xulu, K., Sommer, J., Hinsberger, M., Elbert, T., Weierstall, R., Kaminer, D., Malan-Müller, S., Seedat, S., & Hemmings, S. (2020). P.698 DNA methylation correlates of Narrative Exposure Therapy for Forensic Offender Rehabilitation in trauma-exposed men with appetitive aggression. *European Neuropsychopharmacology*, *40*, S397–S398. <https://doi.org/10.1016/j.euroneuro.2020.09.516>

Zang, Y., Hunt, N., and Cox, T. (2013). A randomised controlled pilot study: the effectiveness of narrative exposure therapy with adult survivors of the Sichuan
earthquake. *BMC Psychiatry* 13:41. doi: 10.1186/1471-244x-13-41

Zang, Y., Hunt, N., and Cox, T. (2014). Adapting narrative exposure therapy for Chinese earthquake survivors: a pilot randomised controlled feasibility study. *BMC Psychiatry* 14:262. doi: 10.1186/s12888-014-0262-3

Zolfa, R., Moradi, A., Mahdavi, M., Parhoon, H., Parhoon, K., and Jobson, L. (2022). Feasibility and acceptability of written exposure therapy in addressing posttraumatic stress disorder in Iranian patients with breast cancer. *Psycho-Oncology* 32, 68–76. doi: 10.1002/pon.6037
